# Superhydrophilic Fe^IV^_2_Mn^II^ Nanocluster: A Combined Diagnostic and Therapeutic Agent

**DOI:** 10.1002/anie.202512874

**Published:** 2025-10-07

**Authors:** Ye Xu, Chaojie Tang, Guang Deng, Chao Li, Zhaoyang Liu, Zhen‐Feng Chen, Xiaoyu Wu, Wu Wang, Zhao‐Xi Wang, Jonathan L. Sessler

**Affiliations:** ^1^ Department of Chemistry Center for Supramolecular Chemistry and Catalysis Innovative Drug Research Center Shanghai University Shanghai 200444 China; ^2^ Institute of Molecular Medicine (IMM) Shanghai Key Laboratory for Nucleic Acid Chemistry and Nanomedicine Renji Hospital School of Medicine Shanghai Jiao Tong University Shanghai 200240 China; ^3^ Department of Diagnostic and Interventional Radiology Shanghai Six People's Hospital School of Medicine Shanghai Jiao Tong University Shanghai 200233 China; ^4^ TomeWave Suzhou Medical Imaging Co., Ltd Suzhou 215425 China; ^5^ Shanghai Key Laboratory of Rare Earth Functional Materials Department of Chemistry and Materials Science Shanghai Normal University Shanghai 200234 China; ^6^ Key Laboratory for Chemistry and Molecular Engineering of Medicinal Resources (Ministry of Education of China) Guangxi Key Laboratory of Chemistry and Molecular Engineering of Medicinal Resources School of Chemistry and Pharmaceutical Sciences Guangxi Normal University Guilin 541004 China; ^7^ Department of Radiology Longhua Hospital Shanghai University of Traditional Chinese Medicine Shanghai 200032 China; ^8^ Department of Chemistry The University of Texas at Austin 105 East 24^th^ Street, Stop A5300 Austin Texas 78712‐1224 USA

**Keywords:** Iron(IV) nanocluster, Photothermal therapy, Superhydrophilicity, Theranostic agents

## Abstract

We report here an atomically precise nanocluster, (NH_4_)_2_Mn^II^(H_2_O)_2_[Fe^IV^(L)]_2_·11H_2_O (**Fe_2_Mn**). This system was designed to harness superhydrophilic interactions to enhance magnetic resonance imaging (MRI), while permitting photoacoustic imaging (PAI) and photothermal antitumor therapy. It was synthesized using Fe‐HDCL, an Fe(IV) system with strong near infrared (NIR) photothermal conversion capabilities, as a precursor and manganese(II) ion that allows for MRI as the metal center. **Fe_2_Mn** not only produces a useful photothermal effect but also provides for MRI enhancement with a longitudinal relaxivity (*r*_1_) of 7.67 mM^−1^s^−1^ at 0.5 T and 6.02 mM^−1^s^−1^ at 3.0 T, values that are nearly double those of common gadolinium‐based contrast agents (e.g., Gd‐DTPA, *r*_1_ ≈ 3.0 ∼ 4.0 mM^−1^s^−1^). This enhanced relaxivity arises from two *para*‐positioned water coordination sites (*q* = 2) on the manganese(II) ion and robust hydrogen bonding between hydrophilic groups on the Fe‐HDCL surface and surrounding water molecules, forming a second‐sphere hydration shell. As detailed below, the nanocluster of this study (**Fe_2_Mn**) enables high‐quality dual‐mode MR/PA imaging and effective low‐dose (25 µmol·kg^−1^) laser‐triggered photothermal tumor ablation. The present study thus demonstrates how rationally designed superhydrophilic architectures that control both inner sphere coordination and second sphere hydration dynamics can give rise to more effective theranostic agents.

## Graphical abstract

An atomically precise nanocluster with a superhydrophilic architecture acts as an efficient magnetic resonance imaging (MRI) and photoacoustic imaging (PAI) dual‐mode diagnostic agent, while providing for a photothermal‐based antitumor effect.

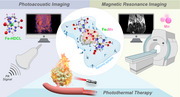

## Introduction

Magnetic resonance imaging (MRI) is a powerful noninvasive imaging method that is widely used in the clinic.^[^ ^1^, ^2^, ^3^ ^]^ In many instances the effectiveness of MRI can be enhanced through the use of a contrast agent. Because the gadolinium(III) ion exhibits long electronic relaxation times due to its seven unpaired electrons,^[^ ^4^ ^]^ it emerged early on as the standard for MRI contrast agent development.^[^ ^5^ ^]^ To stabilize the metal center and reduce toxicity, early MRI contrast agents consisted of complexes wherein the Gd(III) cation was chelated by aminopolycarboxylic acid ligands,^[^ ^6^ ^]^ such as, gadopentetate acid dimeglumine (Gd‐DTPA).^[^ ^1^ ^]^ Unfortunately, these first generation Gd complexes were found in certain cases to trigger a potentially fatal disease known as nephrogenic systemic fibrosis (NSF) in patients who suffer severe renal failure.^[^ ^7^ ^]^ Although macrocyclic Gd(III) complexes continue to see use, there is an incentive to develop alternative MRI contrast agents. The Mn(II) cation has emerged as attractive in this regard.^[^ ^8^ ^]^ Manganese is an essential element in human biology and in its high spin divalent state is typically characterized by five unpaired electrons.^[^ ^9^ ^]^ As a result, several manganese(II)‐based complexes are being studied as alternatives to gadolinium in the context of MRI; however, their limited relaxivity remains a critical barrier for their application in sensitive detection.^[^ ^10^, ^11^, ^12^, ^13^ ^]^

Based on the Solomon–Bloembergen–Morgan (SBM) theory, strategies for enhancing relaxivity of MRI contrast agents are well known. Typically, they involve increasing the number of bound water molecules (*q*), prolonging the rotational correlation time (*τ*_R_), and optimizing the water residence time (*τ*_M_).^[^ ^14^, ^15^ ^]^ Modulating the number and contribution of inner‐sphere water molecules., i.e., those directly bound to a paramagenetic metal ion, is a time‐honored approach.^[^ ^16^, ^17^, ^18^ ^]^ However, for the design of high‐performance MRI contrast agents it is helpful to control second‐sphere water molecules, namely those that can interact with the ligands and the paramagnetic metal center through, e.g., hydrogen‐bond interactions. Indeed, it has been shown that introducing surface ligands with strong water interactions on nanoparticles can enhance relaxivity.^[^ ^19^ ^]^ Therefore, we sought to develop a system that would synergistically overcome intrinsic relaxivity constraints through both inner‐ and second‐sphere contributions. Here, we report a water soluble heterometallic trinuclear nanocluster, (NH_4_)_2_Mn^II^(H_2_O)_2_[Fe^IV^(L)]_2_·11H_2_O (**Fe_2_Mn**), engineered through dual‐sphere optimization to act as an MRI agent while serving concurrently as a photothermal therapy (PTT) sensitizer and allowing photoacoustic imaging (PAI). **Fe_2_Mn** is thus a so‐called theranostic, an agent that permits concurrently both therapy and imaging.^[^ ^20^, ^21^, ^22^ ^]^ In the present instance, imaging is achieved through two different diagnostic modalities, namely MRI and PAI.

The integration of multiple imaging modalities constitutes a promising strategy to overcome the inherent limitations of individual techniques.^[^ ^23^, ^24^, ^25^ ^]^ MRI remains a cornerstone of clinical diagnostics due to its unlimited tissue penetration and exquisite soft‐tissue contrast, which permits detailed anatomical visualization of deep‐seated lesions. However, its low sensitivity can limit its utility.^[^ ^26^, ^27^ ^]^ Conversely, PAI has emerged as a highly sensitive modality that leverages laser‐induced thermoelastic expansion to provide functional and molecular information, particularly of superficial vascular structures.^[^ ^28^, ^29^, ^30^ ^]^ Nevertheless, the penetration depth of PAI remains constrained to a few centimeters, limiting its utility for deep‐tissue applications.^[^ ^29^ ^]^ The complementary nature of these techniques makes their combination attractive: MRI provides the anatomical roadmap for deep tissues, while PAI adds highly‐sensitive functional imaging at more superficial depths. This synergy is especially valuable in therapeutic applications, where MRI can guide treatment planning to deep tumor margins while PAI offers real‐time monitoring of photothermal therapy efficacy.

Currently, the majority of systems that achieve dual‐modal MR and PA imaging are nanoplatforms.^[^ ^31^ ^]^ Unfortunately, they carry the risk of undesirable intracellular catabolism and long‐term retention after intravenous administration;^[^ ^32^, ^33^, ^34^ ^]^ this can give rise to regulatory concerns.^[^ ^35^, ^36^ ^]^ In fact, contrast agents that permit both enhanced MR and PA imaging are rare, especially those with acceptable toxicity profiles.^[^ ^37^, ^38^, ^39^ ^]^ Fortunately, recent studies have established that some atomically precise nanoclusters with diameters of 1–3 nm and well‐defined structures display good biocompatibility and can be eliminated efficiently from mice through hepatic and renal excretion with no evidence of toxicity.^[^ ^40^, ^41^ ^]^

**Scheme 1:**
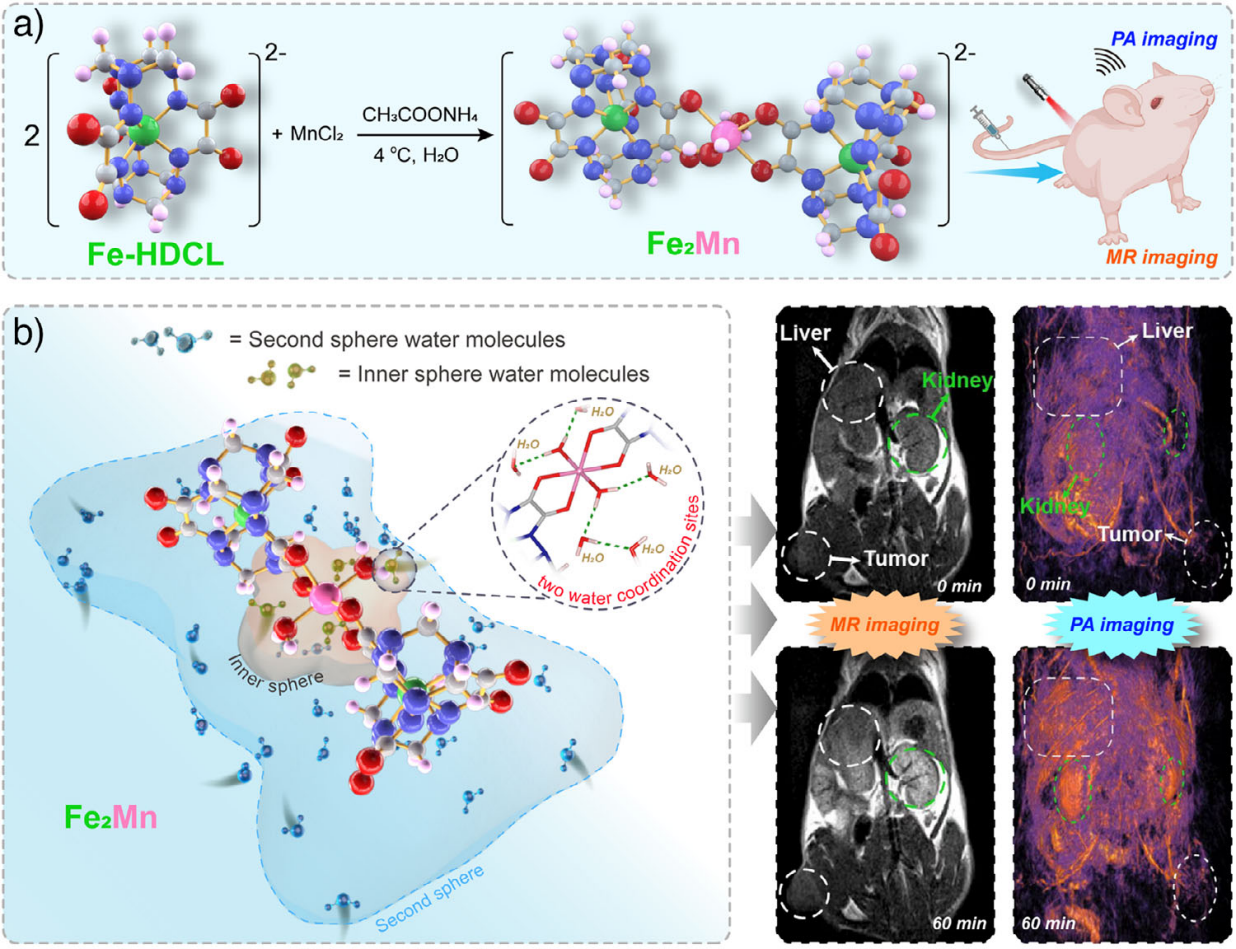
Schematic illustration showing the preparation of **Fe_2_Mn** a) and its proposed used in MR and PA imaging b).

Our previously reported Fe‐HDCL was identified as a promising agent for both PAI and PTT because of its strong NIR photothermal conversion.^[^ ^42^ ^]^ However, it lacks MRI functionality. On the other hand, its numerous hydrophilic groups inspired us to explore its potential as a ligand that when combined with a paramagnetic metal ion would create a putative high‐performance MRI contrast agent. With such considerations in mind, we synthesized a discrete trinuclear heterometallic nanocluster (**Fe_2_Mn**). The preparation was carried out in aqueous solution using Fe‐HDCL as a precursor in combination with a manganese(II) ion. The resulting complex (**Fe_2_Mn**), containing both Fe(IV) and Mn(II) ions, not only exhibited a photothermal effect similar to Fe‐HDCL, it also serves as an effective PAI and PTT agent. As importantly, **Fe_2_Mn** was found to act as an MRI enhancer, with a longitudinal relaxivity (*r*_1_) of 7.67 mM^−1^s^−1^. This value is nearly twice that of gadolinium‐based contrast agents in clinical use (e.g., Gd‐DTPA, *r*_1_ ≈3.0 ∼4.0 mM^−1^s^−1^). In fact, to our knowledge, **Fe_2_Mn** is the strongest monodispersed Mn‐based MRI contrast agent reported to date. This atomically precise theranostic agent demonstrates high biocompatibility, rapid clearance, and passive tumor targeting following low‐dose administration, with its high relaxivity stemming from a synergistic interplay between para‐coordinated inner‐sphere waters on the Mn(II) center and a dense second‐sphere hydration shell that is supported by the superhydrophilic surfaces of the constituent Fe‐HDCL moieties (Scheme 1).

## Results and Discussion

**Figure 1:**
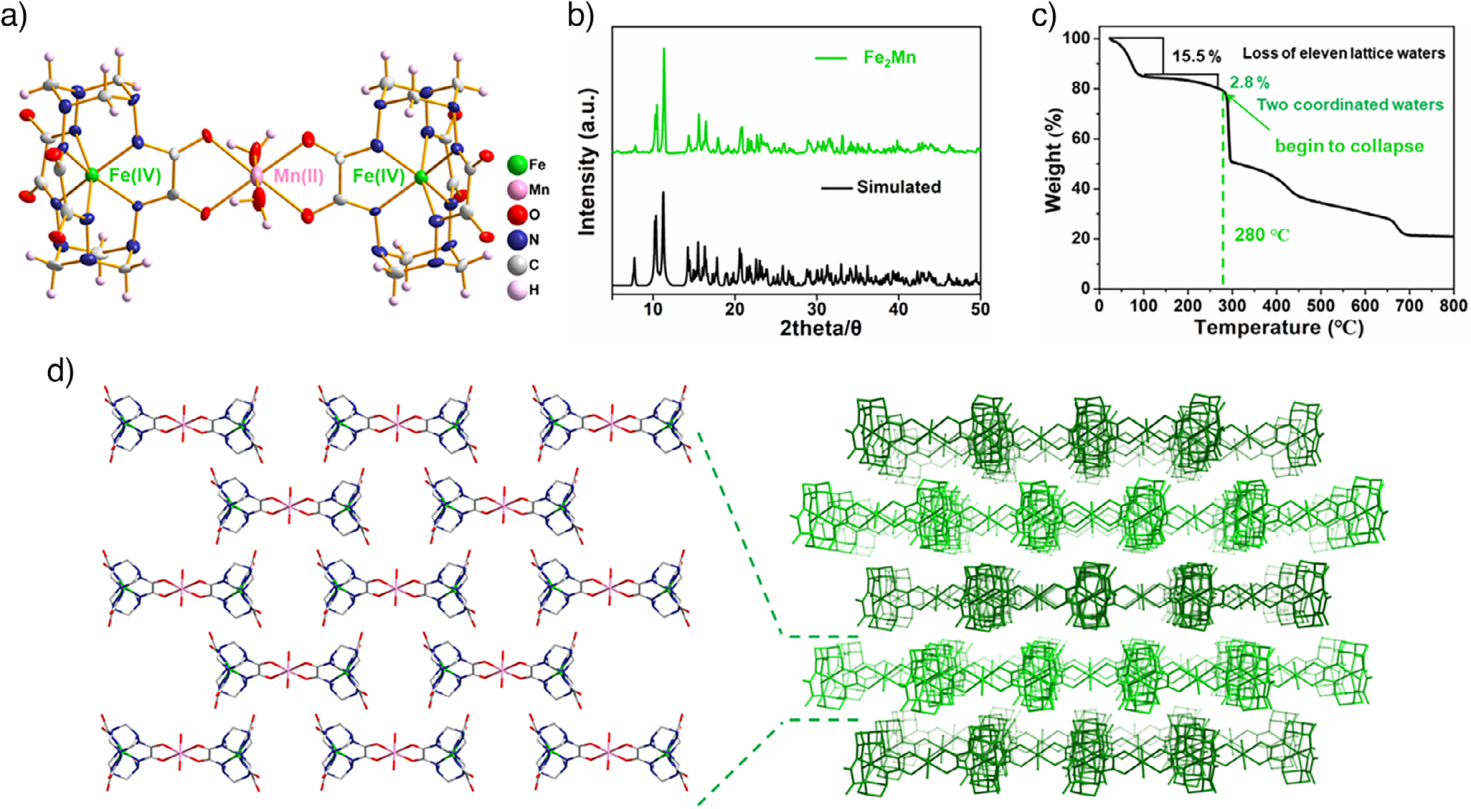
Characterization data for **Fe_2_Mn**. a) Molecular structure. Lattice water molecules are omitted for clearly. b) Powder X‐ray diffraction patterns. c) Thermogravimetric curve. d) View of the 2D and 3D structures of **Fe_2_Mn**.

The synthesis of **Fe_2_Mn** is shown in Scheme 1a. It takes advantage of the fact that Fe‐HDCL presents three sets of *ortho*‐oxalyl‐derived hydrazide oxygen atoms along the *Y*‐axis that are separated by 120 degrees along the *X*‐axis. These constitute open coordination sites that are potentially capable of interacting with other functional metal ions and clusters. To obtain an atomically precise nanocluster, water was selected as the solvent, Mn(II) chloride was employed as the manganese source, and ammonium acetate was used as the buffer medium. Keeping the temperature low (ca. 4 °C) allowed diffraction grade single crystals to be obtained. An X‐ray diffraction analysis then confirmed that the discrete nanocluster **Fe_2_Mn** had been formed. The purity of the bulk material and its correspondence to the single crystals was validated using both elemental analysis and powder X‐ray diffraction studies (Figure 1b).

**Fe_2_Mn** crystallizes in the triclinic $P\bar{1}$ space group (Table S1) and contains two Fe‐HDCL subunits, one manganese ion, two ammonium ions, two coordinated water molecules, and solvent molecules (Figure 1a). The coordination environment and the metric parameters were taken as evidence that the two iron centers were in the Fe(IV) oxidation state as true in Fe‐HDCL.^[^ ^43^ ^]^ The oxidation state of the manganese ion was identified as +2 on the basis of bond‐valence calculations (Table S2). The anionic unit of **Fe_2_Mn** consisted of one six‐coordinate Mn(II)‐coordination unit, in which the Mn(II) ion exists in a distorted octahedral coordination environment. This environment comprises an equatorial plane occupied by four hydrazide oxygen atoms from two Fe‐HDCL moieties and two water molecules bound at the axial positions of the pseudo‐octahedron. Accordingly, the two water bound molecules in **Fe_2_Mn** are in a *para*‐type arrangement. This configuration is relatively rare among reported nanoclusters.^[^ ^44^, ^45^, ^46^ ^]^ Moreover, classic gadolinium‐ and manganese‐based contrast agents with two coordinated water molecules generally display an *ortho*‐type arrangement as the result of being based on polyamine chelating ligands.^[^ ^7^, ^40^ ^]^

To explore the thermal stability of **Fe_2_Mn**, it was subject to heating at a rate of 10 °C min^−1^ from room temperature to 800 °C under a nitrogen atmosphere. As illustrated in Figure 1c, the resulting thermogravimetric curve revealed weight loss corresponding to approximately eleven lattice water molecules over the 30 to 100 °C temperature range and weight loss corresponding to the two coordinated water molecules in the 100 and 260 °C temperature range. **Fe_2_Mn** undergoes further decomposition starting at about 280 °C. This decomposition temperature is higher than what was seen for Fe‐HDCL (250 °C), leading us to suggest that **Fe_2_Mn** possesses better thermal stability than its constituents.

Our previous study served to confirm that Fe‐HDCL can support strong hydrogen bond interactions with adjacent water molecules (Figure S1). Similarly, numerous intermolecular O─H⋯O hydrogen bonding interactions between the **Fe_2_Mn** core cluster and water molecules are observed in the solid state (Table S3). In the case of **Fe_2_Mn**, 2D sheets are stabilized by an apparent hydrogen bond network; however, the sheets remain separated, presumably as the result of steric hinderance (Figure 1d).

**Figure 2:**
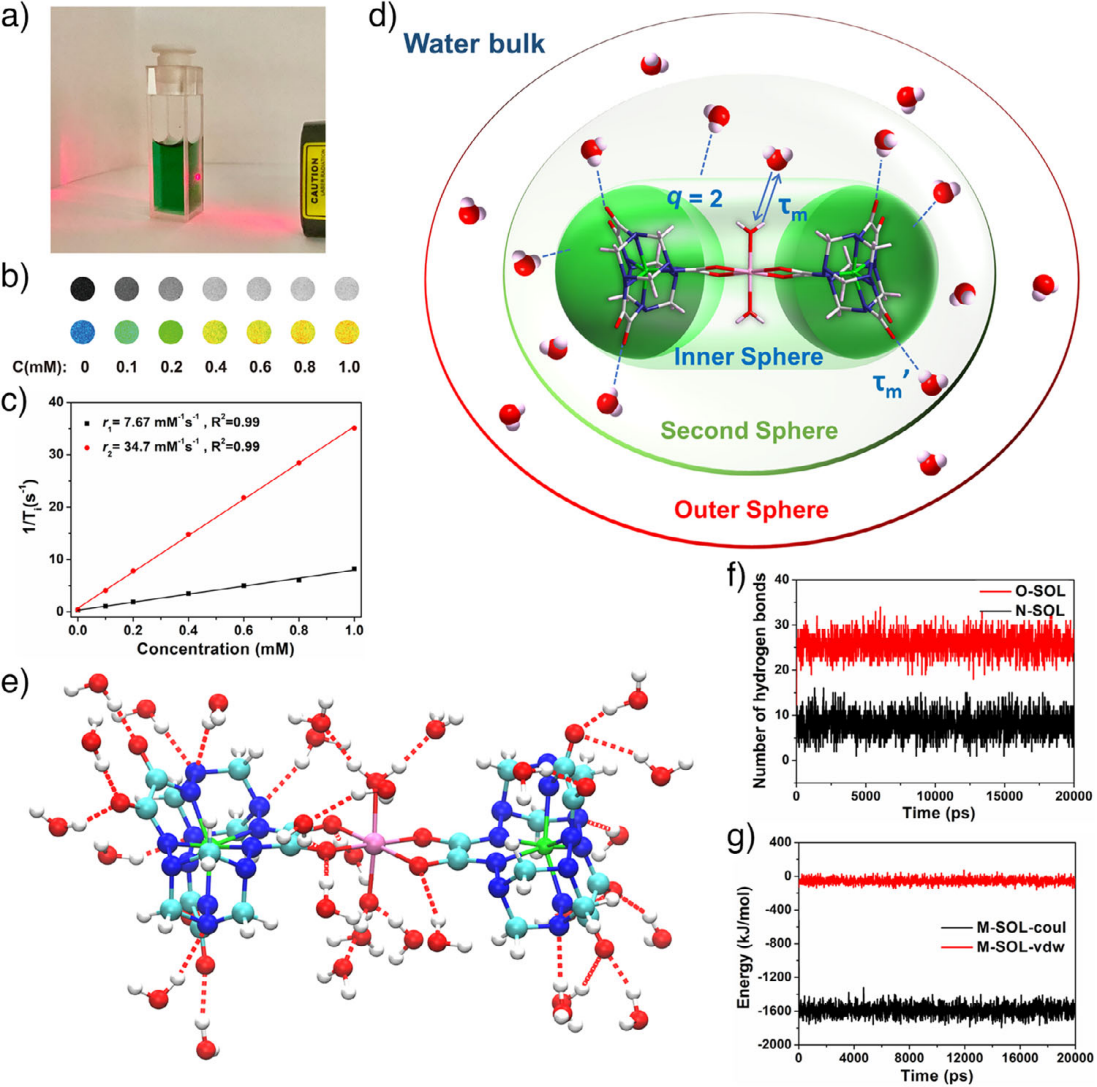
a) Photograph showing the lack of Tyndall effect when **Fe_2_Mn** (100 µM aqueous solution) is subject to photoirradiation. In vitro *T*_1_‐weighted and color‐mapped phantom MR images b) and MR relaxation rates c) of **Fe_2_Mn** at different concentrations under an applied 0.5 T magnetic field at 25 °C. d) Schematic showing the key molecular parameters that are expected to influence the inner and second sphere relaxivity of **Fe_2_Mn**; these include hydration number (*q*) and mean residence time of the bound water (*τ*_m_). (e) Hydrogen bond interactions between one **Fe_2_Mn** nanocluster and proximal water molecules in a water bath at 1 bar and 25 °C per the Berendsen barostat and V‐rescale thermostat, respectively, as calculated by a molecular dynamics (MD) simulation. f) Average number hydrogen bonds formed between O atoms and N atoms in **Fe_2_Mn** and water molecules. g) Energy of the average electrostatic (coul) and van der Waals (vdw) interactions between a **Fe_2_Mn** nanocluster and surrounding water molecules.

**Fe_2_Mn** was found to be soluble in water up to a concentration of 5 mM (cf. Figure S2). This water‐solubility is ascribed to the many hydrophilic groups spread around the nanocluster. When an aqueous solution of **Fe_2_Mn** was irradiated with a monochromatic laser, no optical path was observed indicating an absence of Tyndall effects (Figure 2a). Moreover, the particle size of **Fe_2_Mn** was found to lie below the minimum detection limit of dynamic light scattering spectroscopy. These results were taken as evidence that **Fe_2_Mn** is uniformly distributed in water and exists in discrete, single nanocluster form. Furthermore, the small size (ca. 1.4 nm) of the discrete **Fe_2_Mn** cluster was deemed beneficial for rapid drug dissolution (Figure S3) and elimination. Particles smaller than 6 nm are typically cleared rapidly from the body via the renal clearance pathway. This precludes excessive accumulation and associated potential toxicities.^[^ ^36^ ^]^ Considered in concert, these attributes lead us to suggest that **Fe_2_Mn** would be amenable to biological testing without further modification.

Mn(II)‐containing materials have been explored widely as potential *T*_1_‐weighted MRI contrast agents. The relaxation rates and MR images of pure water and aqueous solutions of **Fe_2_Mn** were thus tested at different concentrations under a 0.5 T magnetic field at room temperature (25 °C). As shown in Figure 2b, MR phantom images of **Fe_2_Mn** in aqueous solution brightened with increasing concentration. By fitting the longitudinal relaxation time (*T*_1_) and transverse relaxation time (*T*_2_), longitudinal and transverse relaxation rates of *r*_1_ = 7.67 mM^−1^s^−1^ and *r*_2 _= 34.7 mM^−1^s^−1^ were calculated for **Fe_2_Mn** with *r*_2_/*r*_1_ = 4.52 < 5 (Figure 2c). At a clinically relevant field strength of 3.0 T, **Fe_2_Mn** maintained an *r*_1_ value of 6.02 mM^−1^s^−1^, demonstrating its effectiveness across different magnetic fields (Figure S4).

The longitudinal relaxation rate of Fe‐HDCL is only 0.0931 mM^−1^s^−1^ (Figure S5), making its contribution to the relaxation rate of **Fe_2_Mn** negligible. Therefore, Mn^2+^ ion plays a predominant role in the enhanced relaxivity of **Fe_2_Mn**. However, the reported *r*_1_ values of small molecule Mn‐based MRI contrast agents are only 1–3 mM^−1^s^−1^.^[^ ^10^, ^11^ ^]^ The longitudinal relaxation rate of unchelated Mn^2+^ ion (i.e., MnCl_2_ dissolved in pure water) was 6.56 mM^−1^s^−1^ as measured with an MRI scanner under a 0.5 T magnetic field (Figure S6). In contrast, the *r*_1_ value of **Fe_2_Mn** is 7.67 mM^−1^s^−1^, and, notably, it is 6.02 mM^−1^s^−1^ at 3.0 T. These values surpasses that of the free Mn^2+^ ion and are nearly twice those of commercially available gadolinium‐based contrast agents (such as, Gd‐DTPA with *r*_1_ values around 3.0–4.0 mM^−1^s^−1^).^[^ ^4^ ^]^ To the best of our knowledge, the *r*_1_ value for **Fe_2_Mn**, which is maintained at clinical field strength (i.e., 3.0 T), represents the highest known for a monodisperse Mn‐based *T*_1_‐weighted MRI contrast agent.^[^ ^47^ ^]^

The high relaxivity of **Fe_2_Mn** arises from the combined contributions of inner‐sphere coordination and second‐sphere hydration. Direct binding of two inner‐sphere water molecules (*q* = 2) to the Mn(II) center provides an important contribution, as relaxivity scales with the number of coordinated water molecules in the inner sphere.^[^ ^11^ ^]^ **Fe_2_Mn** also engages in robust hydrophilic interactions with bulk water to form a hydrogen bond‐stabilized “second water sphere” around the nanocluster. This second sphere acts to increase the local water concentration and prolongs the water residence time, further enhancing the relaxation efficiency. Although increased molecular dimensions can prolong modestly the rotational correlation time (*τ*_R_), in the case of **Fe_2_Mn** this effect is overshadowed by the dominant inner and second sphere mechanisms.^[^ ^48^ ^]^ That high relaxivity is maintained at 3.0 T further provides support for the suggestion that second‐sphere hydration plays a role in mitigating the field‐dependent decrease in relaxivity that would otherwise be expected.

In the solid state, **Fe_2_Mn** exhibits multidirectional hydrophilic interactions with water molecules, a finding that is consistent with the presence of a shell‐like “second water sphere” in solution (Figure 2d).^[^ ^49^ ^]^ This hydration shell wraps around the nanocluster in the crystal structure, creating a high local concentration of water molecules stabilized by hydrogen bonding interactions (Figure S7). Experimental evidence for these strong hydrogen bonds came from FT‐IR spectroscopic analyses. The broad O‐H/N‐H stretching band about 3271 cm^−1^ and a redshifted C═O vibration at 1624 cm^−1^ are characteristic of extensive hydrogen bonding (Figure S8a). The reversible attenuation of these features upon dehydration is taken as evidence for the critical role water molecules play in mediating these interactions (Figure S8b). Further support for this suggestion came from molecular dynamics (MD) simulations performed in a simulated aqueous environment. The results revealed that each **Fe_2_Mn** cluster maintains >35 hydrogen bonds with the surrounding water molecules, significantly exceeding the ancillary bonding seen in the crystalline state. Specifically, per the calculations, the O atoms in **Fe_2_Mn** form an average of 25.8 hydrogen bonds with water, while the N atoms form 10.0 bonds (Figure 2f). The net result is stabilization of a dense hydration shell around the nanocluster, which is proposed to contribute to the effective relaxivity observed at both 0.5 and 3.0 T.

Various electrostatic and van der Waals interactions between **Fe_2_Mn** and water molecules are noted in the calculations. Both sets of interactions are energetically favorable with the average values found to be −1588 and −55.9 kJ/mol, respectively (Figure 2g). Moreover, the equilibrium between the **Fe_2_Mn** cluster and these water molecules is reached almost instantaneously with a stable oscillatory state then being maintained. These results are ascribed to the presence of superhydrophilic areas on the surface of **Fe_2_Mn**, which serve to increase the number of exchangeable water molecules (*q*’) and their residency times (*τ*_M_ and *τ*_M_’) in both the inner and second spheres, as well as the diffusion time (*τ*_D_) of the water molecules in the outer sphere.^[^ ^37^ ^]^ Per the SBM theory, this is expected to increase the paramagnetic relaxation provided by **Fe_2_Mn** and improve the experimentally observable *r*_1_.^[^ ^50^ ^]^

As a control, a heterometallic binuclear nanocluster Mn(H_2_O)_4_[Fe(L)]·8H_2_O (**FeMn**) was synthesized. Its structure is similar to **Fe_2_Mn**, except for the absence of one Fe‐HDCL unit. Notably, **FeMn** supports four coordinated water molecules and exhibits nearly identical longitudinal relaxation rates as does **Fe_2_Mn**, despite having twice the inner‐sphere waters (*q* = 4 versus *q* = 2) (Figure S9). On this basis we suggest that the Fe‐HDCL constituents in **Fe_2_Mn** drive second‐sphere relaxivity enhancement through superhydrophilic surface effects. These then synergize with the Mn(II) center to produce the high r_1_ values observed by experiment.

The development of high‐relaxivity magnetic resonance contrast agents has long been constrained by a fundamental trade‐off: while increasing inner‐sphere water coordination (*q*) enhances proton relaxation, it simultaneously destabilizes metal complexes through entropic penalties and ligand‐field weakening.^[^ ^51^ ^]^ This paradox explains why clinically approved gadolinium‐based agents rarely have *q* values that exceed 2. Against this backdrop, the **Fe_2_Mn** nanocluster represents a potential advance. Its anionic architecture, featuring Mn(II) rigidly encapsulated between two Fe‐HDCL subunits with *para*‐positioned water ligands, confers thermodynamic stability that is not seen in conventional bishydrated systems (i.e., those with *q* = 2). Control experiments highlight this advantage: For example, the neutral binuclear analogue **FeMn** (*q* = 4) precipitates within 24 h in aqueous solution (Figure S10), whereas **Fe_2_Mn** remained fully soluble for >30 days (Figure S11).

The stability seen for **Fe_2_Mn** was recapitulated in various biological media. Continuous incubation in normal saline (NS), phosphate buffered saline (PBS), and Dulbecco's modified eagle medium (DMEM) over 48 h led no detectable structural degradation, with relaxivity values remaining stable over the 5.2 to 7.4 pH range (Figure S12). Competitive ligand assays using Eriochrome Black T failed to reveal the presence of free Mn^2+^, mitigating potential metal leakage concerns (Figure S13). The stability of **Fe_2_Mn** was expected to allow its safe use, a supposition that was tested in vitro using a mouse breast cancer cell line (4T1) and a human umbilical vein endothelial cell line (HUVEC) with monitoring provided by a standard methyl thiazolyl tetrazolium (MTT) assay. Solutions of **Fe_2_Mn** of varying concentrations were added dropwise to the HUVEC and 4T1 cells, which were then cultured for either 12 h or 24 h. Cell viability in both cell lines proved to be near 100% after 24 h incubation even at the highest concentration of **Fe_2_Mn** (200 µM) (Figure S14). In contrast, the free divalent manganese ion (incubated as the chloride salt) displayed considerable toxicity and led to cell death even at low concentrations (Figure S15).

For clinical use, a contrast agent should be excreted effectively once a desired image is recorded. To test the excretion features of **Fe_2_Mn**, three BALB/c mice were subject to intravenous injection of a PBS solution of **Fe_2_Mn** at a 25 µmol·kg dose. The dark green urine from the mice was collected within 120 min after injection and analyzed by UV–vis‐NIR absorption spectroscopy (Figure S16). This analysis revealed the same spectral features seen for an aqueous solution of **Fe_2_Mn**, leading us to conclude that it is cleared relatively rapidly from the body after injection into mice and that it remains stable over the time period involved. To quantify further the biodistribution and clearance of **Fe_2_Mn**, we performed ICP‐MS analyses of major organs (liver, kidneys, and spleen) from mice at 24 and 48 h postinjection. The Mn and Fe levels in these organs showed no statistically significant difference from those in untreated control mice at both time points (Figure S17). This was taken as evidence of rapid and complete systemic clearance without residual accumulation. Moreover, hematological analysis and histological examination (H&E staining) of major organs, revealed no signs of systemic toxicity, organ damage, or pathological changes. These findings provide further support for the inferred in vivo stability of the Fe(IV) centers and the biocompatibility of **Fe_2_Mn** (Table S4, Figure S18). We thus suggest that **Fe_2_Mn** may have a role to play as a stable, biocompatible, and readily eliminated theranostic agent.

**Figure 3:**
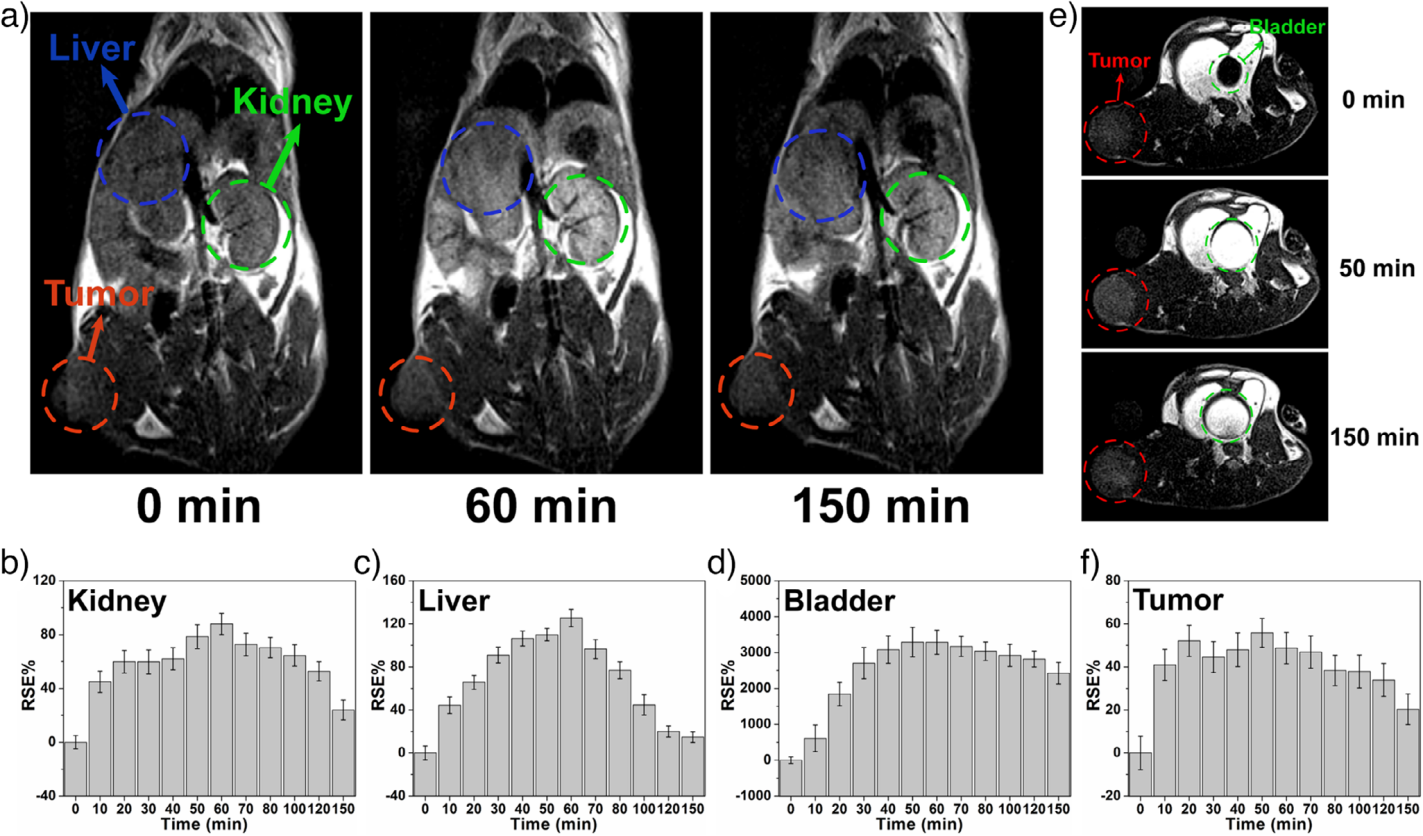
a) In vivo coronal *T*_1_‐weighted MR images of a BALB/c mouse bearing 4T1 tumor xenografts before and after intravenous injection of **Fe_2_Mn** (25 µmol·kg^−1^) at 3.0 T. Relative signal enhancement (RSE %) in kidney b), liver c), bladder d), and tumor (f) at different time points following intravenous injection of **Fe_2_Mn** (25 µmol·kg^−1^). Error bars, mean ± SD (*n* = 3). e) Axial *T*_1_‐weighted MR images of a BALB/c mouse bearing 4T1 tumor xenografts before and after intravenous injection of **Fe_2_Mn** (25 µmol·kg^−1^).

Next *T*_1_‐weighted MR images of BALB/c mice bearing xenograft 4T1 tumors were collected under a 3.0 T magnetic field at various time points after intravenous injection of **Fe_2_Mn** (25 µmol·kg^−1^). As shown in Figure 3a–d, statistically significant enhancement of the MRI signals in the kidney, bladder, and liver was observed at ca. 60 min postinjection. A notable change in the signal at the tumor site was also seen 10 min after injection matching what was inferred from the PA images (cf. Figure S19 and discussion below). The MRI signal intensity gradually increased over time, accompanied by a progressive sharpening of the tumor margins. This improved boundary definition was particularly evident between 10 and 50 min postinjection, coinciding with the period of optimal contrast agent accumulation in the tumor region. As can be seen in Figure 3e,f a maximum relative signal enhancement of approximately 60% was seen for the tumor region ca. 50 min postinjection, after which the enhancement began to diminish gradually. Nevertheless, approximately 20% relative enhancement was still observed ca. 150 min after injection. Compared to Mn‐HDCL (*r*_1_ = 0.357 mM^−1^s^−1^), a manganese analogue of Fe‐HDCL studied as a control, **Fe_2_Mn** was found to provide clearer images; presumably, this reflects its greater relaxivity compared to this previously reported monomeric complex.^[^ ^52^ ^]^ Furthermore, the size of **Fe_2_Mn** is nearly twice that of Mn‐HDCL, resulting in slower clearance in vivo and longer retention times.

**Figure 4:**
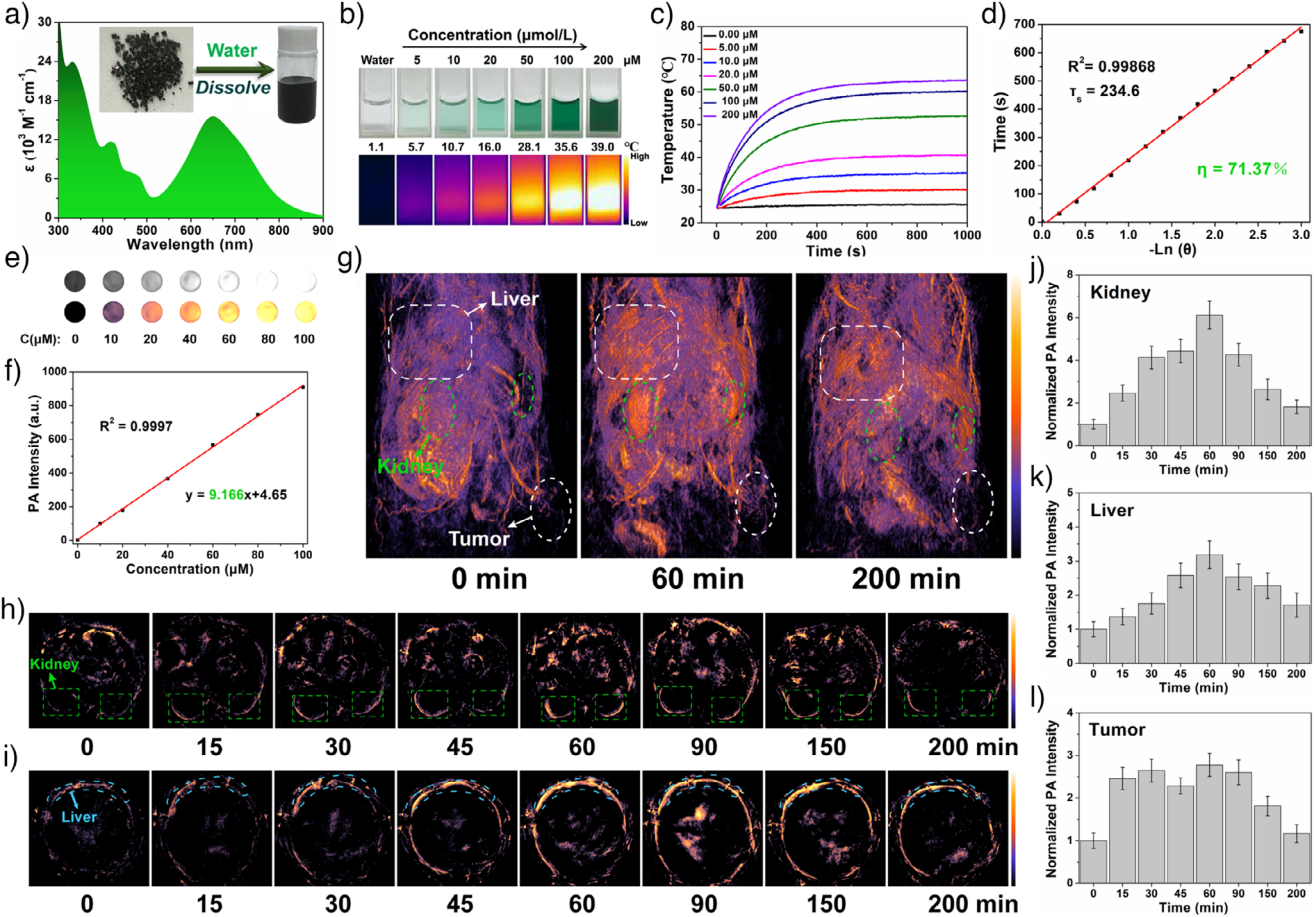
a) UV–vis‐NIR absorption spectra in aqueous solution. The insert shows photographs of crystalline samples and aqueous solutions of **Fe_2_Mn**. b) Optical photographs and thermal images recorded under 730 nm photoirradiation (1.0 W· cm^2^). c) Photothermal curves produced for samples of pure water and different aqueous concentrations of **Fe_2_Mn** upon 730 nm laser photoirradiation (1.0 W· cm^2^). d) Plots of the cooling time versus the negative natural logarithm of the driving force temperature (*θ*) for samples of **Fe_2_Mn** (200 µM). In vitro raw and pseudocolor rendered PA images e) and PA signal intensities f) of **Fe_2_Mn** at different concentrations under 730 nm laser photoirradiation. g) 3D PA images of BALB/c nude mice bearing a xenograft 4T1 tumor at various time intervals following intravenous injection of **Fe_2_Mn** (25 µmol· kg^−1^). Axial PA images of kidney h) and liver i) before and after intravenous injection of **Fe_2_Mn**. The green square and blue strip indicate the kidney and the liver, respectively. Photoirradiation was carried out during data collection by means of a 730 nm laser (180 mJ·cm^−2^). Normalized PA signal intensities in kidney j), liver k), and tumor l) as seen at various time points following intravenous injection of **Fe_2_Mn** (25 µmol·kg^−1^). Error bars, mean ± SD (*n* = 3).

As prepared, **Fe_2_Mn** is produced in the form of dark black crystals that are opaque to high‐intensity visible light (Figure S20). As expected for a dark material, the UV–vis spectrum of **Fe_2_Mn** in the solid state revealed a broad absorption band from 200 to 1000 nm (Figures S21 and S22). Importantly, the solid form of **Fe_2_Mn** absorbs strongly in the near‐infrared spectral region, where tissues display relatively greater transparency. In deionized water at room temperature, the absorption spectrum of **Fe_2_Mn** is characterized by two intense absorption bands in the 300–500 and 550–900 nm spectral region with maxima at 300 and 650 nm, respectively (Figure 4a). The extinction coefficients of the 420, 650, and 730 nm maxima were calculated to be 11700, 15560, and 9820 M^−1^ cm^−1^.

**Fe_2_Mn** was expected to recapitulate the desirable photothermal properties of its precursor, Fe‐HDCL notwithstanding the presence of a coordinated Mn^2+^ center. As a test of this proposition, aqueous solutions of **Fe_2_Mn** at different concentrations were placed in cuvettes. The temperature changes were recorded as a function of time under continuous irradiation with a 730 nm laser (1.0 W·cm^−2^) for 10^3^ s. As shown in Figure 4c, the temperature of pure water, studied as a control, only increased by 1.1 °C from room temperature of 24.5 °C when irradiated for 10^3^ s. In contrast, the temperature of the **Fe_2_Mn** solutions were seen to increase in a concentration dependent manner as inferred from both thermal imaging and direct measurements (cf. Figure 4b,c). For the 200 µM **Fe_2_Mn** sample, an increase to 39.0 °C was observed. The photothermal conversion efficiency (PCE) of this latter sample was determined by recording the temperature changes under 730 nm laser excitation at a power density of 1.0 W·cm^−2^ for 10^3^ s followed by turning off the light source and allowing the sample to cool to room temperature (Figure S23). The heat transfer time constant (*τ*_s_) was determined to be 235 s from a linear plot of the cooling time versus the negative natural logarithm of the driving force temperature (θ) (Figure 4d). Using Roper's method and this *τ*_s_, a PCE value of 71.4% was obtained.^[^ ^53^ ^]^ That this high level of PCE is retained is taken as evidence that the Fe(IV) centers that promote the observed photothermal effects remain electronically unperturbed upon cluster formation. Note: The choice of 730 nm as the irradiation wavelength reflects the greater light penetration into tissues at 730 than at 650 nm (the long‐wavelength λ_max_ of **Fe_2_Mn**). This wavelength mismatch notwithstanding, the photothermal efficiency of the 200 µM **Fe_2_Mn** sample was found to exceed that of other photothermal materials reported in the literature (Table S5). Given that cancer cells display greater heat sensitivity than normal cells,^[^ ^54^ ^]^ the above results prompted us to explore whether **Fe_2_Mn** might have a role to play in photothermal therapy.

A key predicate for use an agent to trigger PTT or to enhance PAI is photostability. Accordingly, the UV–vis‐NIR spectra of aqueous **Fe_2_Mn** samples containing varying concentrations were recorded before and after irradiation with a 730 nm laser (1.0 W·cm^−2^) for 10^3^ s. No evident changes in the absorbance features were seen for any of the samples (cf. Figure S24a). In addition, the temperature variation produced by **Fe_2_Mn** (200 µM aqueous solution) was monitored through eight cycles of laser irradiation for 10^3^ s followed by natural cooling to room temperature. No significant drop in the maximum temperature was seen through multiple cycles (Figure S24b). This was taken as further evidence that **Fe_2_Mn** is photostable.

As a first test of the PAI properties of **Fe_2_Mn,** aqueous solutions of both Fe‐HDCL and **Fe_2_Mn** at different concentrations were irradiated with a 730 nm pulsed laser, with the resulting signal intensities being recorded using a photoacoustic instrument (TomoWave Laboratories, LOIS‐3D China). As shown in Figure 4e, the signal intensity of the **Fe_2_Mn** sample was approximately twice that of the corresponding Fe‐HDCL sample at an identical concentration.^[^ ^42^ ^]^ This doubling is ascribed to the presence of two Fe‐HDCL subunits within each **Fe_2_Mn** nanocluster. As expected, a liner relationship between the **Fe_2_Mn** concentration and the PA signal intensity was seen (Figure 4f).

To probe further the PAI potential of **Fe_2_Mn**, in vivo PAI experiments were performed in BALB/c nude mice bearing xenograft 4T1 tumors. The PA signals of the mice were monitored at different time points before and after intravenous injection of **Fe_2_Mn** (25 µmol.kg^−1^) using the same equipment as above. The acquired signals were then processed to produce high‐resolution 3D PA images using the open‐source 3D Slicer software. As shown in Figure 4g, the blood vessels were more readily visualized in the PA images recorded after **Fe_2_Mn** injection compared to what was seen prior to treatment. Significant enhancement of the PA signals was also observed in the kidney and liver (Figure 4h,i). The PA intensity in the kidney and liver reached its maximum at ca. 60 min postinjection, increasing to ca. 6‐ and 3‐times the initial values, respectively (Figure 4j,k). By 200 min postinjection, the PA intensities of the kidney and liver had diminished to values that were ca. 2‐fold and 1.5‐fold higher than the preinjection values, respectively.

Next, **Fe_2_Mn** was employed for PA diagnostic visualization of BALB/c nude mice bearing xenograft 4T1 tumors. As illustrated in Figure 4l, only weak cancer‐associated PA signals were observed prior to tail vein injection of **Fe_2_Mn** (25 µmol kg^−1^). This lack of effective baseline imaging likely reflects the presence of thin blood vessels within the tumor, which are unfavorable for PA imaging. In contrast, a ca. 2.5‐fold increase in the PA tumor intensity was seen 15 min postinjection with **Fe_2_Mn**. The intensity gradually increased over time, reaching a maximum of 3x baseline ca. 60 min postinjection. Subsequently, the signal began to gradually weaken until it returned to baseline at 200 min postinjection. The highest relative PA signal intensity observed following **Fe_2_Mn** injection was similar to that of Fe‐HDCL at the tumor site, both being ca. 3‐fold higher than the preinjection level. However, **Fe_2_Mn** maintained a 2.5‐fold enhancement over baseline level for roughly 50% longer than Fe‐HDCl (90 versus 60 min) under otherwise identical conditions.^[^ ^42^ ^]^

**Figure 5:**
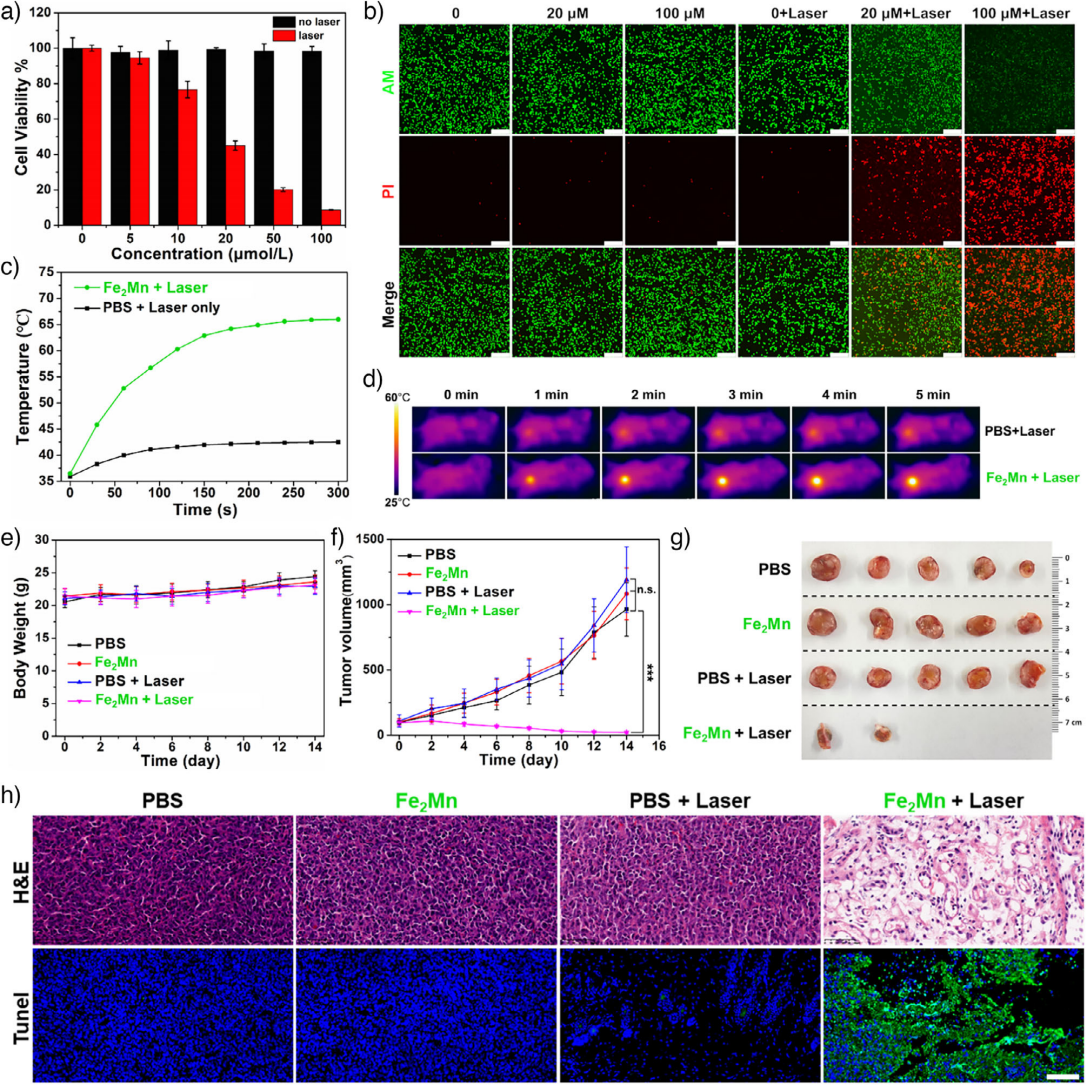
a) Cytotoxicity assays of 4T1 cells incubated with different concentrations of **Fe_2_Mn** with and without 730 nm laser irradiation (1.0 W·cm^−2^, 5 min). b) Fluorescence microscopic images of 4T1 cells (green represents living cells, red represents dead cells) incubated with **Fe_2_Mn** at different concentrations for 12 h and then subject to 730 nm laser irradiation for 5 min at a power density of 1.0 W·cm^−2^. Scale bar: 100 µm. c) Temperature of the tumor sites as a function of irradiation time (730 nm, 0.75 W·cm^−2^). d) Thermal images of BALB/c mice bearing 4T1 tumors after intravenous injection of PBS (100 µL) or **Fe_2_Mn** (25 µmol·kg^−1^) and photoirradiation using a 730 nm laser (0.75 W·cm^−2^). Body weight e) and tumor volumes f) of mice in different groups after laser irradiation. g) Optical photographs of the excised tumors for each group of 5 mice after a 14‐day observation period. Results are expressed as the mean ± SD., *n* = 5/group; n.s.: not significant; statistical significance (or lack thereof) was calculated via a Student's *t*‐test ***p < 0.001. h) H&E and TUNEL staining images of tumor slices collected from the different groups at the end of treatment. Scale bar: 50 µm.

As an initial test of whether the thermal effects (heating) seen upon photoirradiation would translate into controlled toxicity effects, 4T1 cells were incubated with different concentrations of **Fe_2_Mn** before being irradiated with a 730 nm laser (1.0 W·cm^−2^) for 5 min. A cell viability of nearly 100% was seen after 5 min of 730 nm laser irradiation in the absence of **Fe_2_Mn**. In contrast, and as shown in Figure 5a, a dosage‐dependent decrease in cell viability was seen upon incubation with **Fe_2_Mn** with 8.7% cell viability being seen at a concentration of 100 µM. No obvious toxicity was seen for cells treated with **Fe_2_Mn** in the absence of laser irradiation.

To evaluate further the photothermal therapeutic effect of **Fe_2_Mn**, 4T1 cells were monitored by a series of live/dead cell assays using calcein AM and PI staining. Here, live and dead cells give rise to green and red fluorescence, respectively. As shown in Figure 5b, a bright green fluorescence was observed after treatment with different concentrations of **Fe_2_Mn** in the absence of laser irradiation. The same was true for the cells that were not incubated with **Fe_2_Mn** but subject to irradiation (730 nm laser, 1.0 W·cm^−2^, 5 min). In contrast, few of the 4T1 cells treated with **Fe_2_Mn** and subject to 730 nm laser irradiation survived; presumably, this reflects an efficient transformation of light energy into a thermal effect resulting in cell death. A concentration dependence was seen and at 100 µM **Fe_2_Mn** few living cells are observed.

To test the in vivo antitumor efficiency of **Fe_2_Mn** as a PTT agent, 20 4T1‐tumor‐bearing BALB/c mice were divided into four groups (five mice per group). The groups included a control group (PBS), in which mice were only injected with PBS, a “Fe_2_Mn” group, in which mice were injected with **Fe_2_Mn** alone, a “PBS + Laser” group, in which mice were treated with PBS and subject to laser irradiation, and a “Fe_2_Mn + Laser” group, in which mice were treated with **Fe_2_Mn** and subject to NIR laser irradiation. As illustrated in Figure 5c,d, 10 min after tail vein injection of **Fe_2_Mn** (25 mmol·kg^−1^), the tumor temperature had increased to above 66 °C when subject to continuous 730 nm laser irradiation at a power density of 0.75 W·cm^−2^ for 5 min. By contrast, the tumor temperature in the “PBS + Laser” group only reached 42.5 °C under the same conditions.

To evaluate quantitatively the photothermal therapeutic efficacy of **Fe_2_Mn** in vivo, the extent of tumor growth delay was monitored every 2 days for 14 days. Body weights were also recorded. During this treatment period, no significant body‐weight losses were seen for any of the groups (Figure 5e). On the other hand, tumor growth was suppressed within the first 6 days in the “Fe_2_Mn + Laser” group compared with the other three groups (Figure 5f). The tumor volume of the other three groups grew rapidly and at similar tumor growth rates, revealing that neither laser irradiation alone nor the presence of **Fe_2_Mn** in the absence of photoillumination had an appreciable effect on tumor growth. At the end of the monitoring period, all mice were sacrificed, and the resected tumors were photographed. As can be seen from inspection of Figure 5g, most of the tumors in the “Fe_2_Mn + Laser” group were ablated and the few that remained were the smallest among those for all groups. Tumor sections were also stained with H&E and terminal deoxynucleotidyl transferase dUTP nick end labeling (TUNEL) to evaluate cell apoptosis and cell proliferation within tumors. Per our design expectations, the most significant apoptosis and proliferation inhibition was observed for the “Fe_2_Mn + Laser” group (Figure 5h). These results thus provide support for the notion that **Fe_2_Mn** can be an effective PTT agent in vivo.

## Conclusion

We report here a discrete trinuclear heterometallic nanocluster (**Fe_2_Mn**) with intrinsic superhydrophilicity that provides for a high level of magnetic resonance imaging (MRI) enhancement. The presence of two *para‐*positioned inner‐sphere waters (*q* = 2) bound directly to the internal Mn(II) center gives rise to effective paramagnetic relaxation, while the superhydrophilicity of the system serves to stabilize >35 second sphere water molecules, prolonging residency times and accelerating proton exchange in a mutually reinforcing manner. This cooperative interplay supports a high longitudinal relaxivity of 7.67 mM^−1^s^−1^ at 0.5 T, and 6.02 mM^−1^s^−1^ at 3.0 T, values that are roughly twice those seen for clinical gadolinium standards. The anionic *para*‐coordination architecture resolves the historical relaxivity‐stability trade‐off, conferring >30‐day aqueous stability with no evidence of metal leakage. Cell proliferation assays confirmed that **Fe_2_Mn** displays high cell viability at 200 µM and rapid renal clearance. The design of **Fe_2_Mn** also harnesses the photothermal properties of its Fe‐HDCL constituents. This permits high‐fidelity PA imaging of tumors, vasculature and organs at low doses (25 µmol·kg^−1^), as well as significant enhancement of *T*_1_‐weighted MR contrast enabling clear tumor delineation. Efficient photothermal ablation of 4T1 tumor cells was demonstrated both in vitro and in vivo when **Fe_2_Mn** was combined with 730 laser irradiation. Based on the results presented here, we believe that **Fe_2_Mn** is a promising multifunctional imaging‐guided PTT agent that warrants further study as a theranostic for tumor diagnosis and treatment. More broadly, this work demonstrates the power of leveraging both inner‐ and second‐sphere water interactions in designing high‐performance theranostics and highlights the synergy between high‐valent Fe(IV) centers and transition metals, as well as their potential use in biomedical applications.

## Conflict of Interests

The authors declare no conflict of interest.

## Supporting information



Supporting Information

Supporting Information

## Data Availability

The data that support the findings of this study are available in the Supporting Information of this article.
