# Supplementary material for: Superhydrophilic FeIV 2MnII Nanocluster: A Combined Diagnostic and Therapeutic Agent
Source: Angew Chem Int Ed Engl. 2025 Oct 7;64(49):e202512874. doi: 10.1002/anie.202512874 (PMC12668307; doi:10.1002/anie.202512874)
Supplement: Supplementary file 1 — Supporting Information [file ANIE-64-e202512874-s002.pdf]

Supporting Information
©Wiley-VCH 2021
69451 Weinheim, Germany

Superhydrophilic $Fe^{IV}_2Mn^{II}$ Nanocluster: A Combined Diagnostic and Therapeutic Agent

Table of Contents

1 Experimental section 3
1.1 Materials and characterization 3
1.2 Synthesis of $(NH_4)_2Mn(H_2O)_2[Fe(L)]_2 \cdot 11H_2O$ (Fe_2Mn) 3
1.3 Synthesis of $Mn(H_2O)_4[Fe(L)] \cdot 8H_2O$ (FeMn) 4
1.4 X-Ray crystallography 4
1.5 Photothermal testing 4
1.6 Cytotoxicity tests 5
1.7 Photothermal therapy in vitro 6
1.8 Confocal laser scanning microscopy 6
1.9 Photoacoustic imaging 6
1.10 Longitudinal relaxation time T_1 and relaxivity r_1 measurements 7
1.11 Molecular Dynamics Simulation 7
1.12 MR imaging in vivo 8
1.13 Photothermal therapy in vivo 8
2. Supplementary Data 9
2.1 Crystallographic data 9
2.2 Bond valence sums 9
2.3 Hydrogen bonding data 10
2.4 Hematological analysis 12
2.5 Photothermal conversion efficiency 13
2.6 Hydrogen bond interactions seen in the solid state 14
2.7 Photographs of samples in aqueous solution 14
2.8 Distance parameters and size calculation for Fe_2Mn 15
2.9 MR relaxivity rate measurements for Fe_2Mn 15
2.10 MR relaxivity rate measurements of Fe-HDCL 16
2.11 MR relaxivity rate measurements for $MnCl_2$ 16
2.12 Interactions between Fe_2Mn and water molecules in the solid state 17
2.13 Solid-state FT-IR spectra of Fe_2Mn. 17
2.14 MR relaxivity rate measurements for FeMn 18
2.15 Stability comparison of FeMn in aqueous solution 18
2.16 Long-term solubility of Fe_2Mn in aqueous solution. 19
2.17 Relaxivity measurements in various media 19
2.18 Free manganese(II) ion measurements with Erichrome Black T. 20
2.19 Cell viability studies involving Fe_2Mn 20
2.20 Cell viability studies for $MnCl_2$ 21
2.21 Biological clearance studies 21
2.22 Biodistribution studies 22
2.23 Pathological examination 22
2.24 MR images of an implanted murine tumor 23
2.25 Photographs of crystalline samples 23
2.26 UV-vis-NIR absorption spectrum of a powder sample of Fe_2Mn 24
2.27 UV-vis-NIR spectrum of Fe_2Mn vs the solar spectrum 24
2.28 Photothermal studies 25
2.29 Stability testing 25
3. Supplementary references 26

1 Experimental section

1.1 Materials and characterization

All chemicals were of reagent grade and used as received from commercial sources without further purification. Infrared spectra were recorded using a Nicolet A370 FT-IR spectrometer with pressed KBr pellets in the 400-4000 cm^{-1} region. Elemental analyses for carbon, hydrogen and nitrogen were performed on a Vario EL III elemental analyzer. UV-vis-NIR absorption spectra were recorded using a Beckman Coulter DU 730 spectrometer. Thermogravimetric analyses (TGA) were carried out with a Netzsch STA 449C thermal analyzer at a heating rate of 10 ºC/min in a nitrogen atmosphere. Powder X-ray diffraction (PXRD) data were collected on a Rigaku D/ Max-2200 diffractometer with Cu Kα radiation (λ = 1.5406 Å) over the 5−50° 2θ range. Changes in solution temperature were measured using an FLIR A300 thermal camera. Actual photographs were taken with an Apple iPhone.

All experimental protocols involving animals were approved by the Ethical Committee on Animals at Shanghai University under license number SCXK (Shanghai) 2018-0004 and certificate number 20180004065033. The BALB/c mice and BALB/c-nude mice used in this study were obtained from the Shanghai jiesijie Laboratory Animal Co., Ltd.

1.2 Synthesis of $(NH_4)_2Mn(H_2O)_2[Fe(L)]_2·11H_2O$ (Fe_2Mn)

Fe-HDCL was synthesized according to our previously reported method.[1]

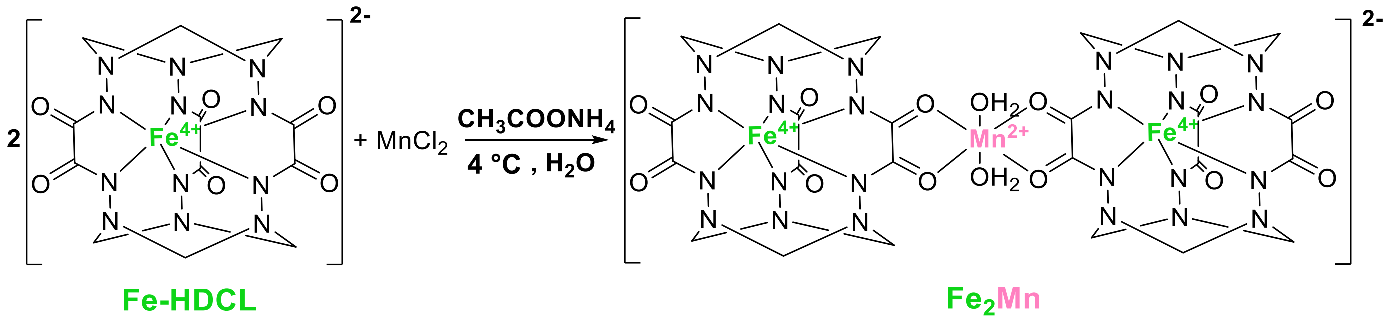

Fe-HDCL (28.41 mg, 0.05 mmol) and ammonium acetate (77.08 mg, 1 mmol) were allowed to stir in 10 mL water until the solid was completely dissolved. Then, $MnCl_2·4H_2O$ (9.90 mg, 0.05 mmol) was added into the mixture and the resulting solution was left to stand in refrigerator. Black block-shaped crystals precipitated from the solution about one week later. Yield 19.28 mg (61.2%). Elemental analysis for $C_{24}H_{59}N_{26}O_{25}Fe_2Mn$ (1278.51): calculated %: C, 22.55; H, 4.651; N, 28.48. Found %: C, 22.89; H, 4.772; N, 29.16. Infrared absorption bands: 3394 cm^{-1}(s) O-H stretch, 3061 (w) C-H stretch, 1603 (vs) C=O stretch, Amide I, 1428 (w), 1378 (w), 1204 (w), 1172 (w), 993 (w), 947 (w), 907 (w), 846 (w), 799 (w), 739 (w), 650 (w).

1.3 Synthesis of $Mn(H_2O)_4[Fe(L)]\cdot 8H_2O$ (FeMn)

Fe-HDCL (28.41 mg, 0.05 mmol) and $MnCl_2\cdot 4H_2O$ (39.60 mg, 0.2 mmol) were each allowed to stir in 10 mL water until the solid was completely dissolved. Then, the solution was left to stand in a refrigerator. Black strip-shaped crystals precipitated from the solution about three days later. Yield 29.89 mg (80%). Elemental analysis for $C_{24}H_{59}N_{26}O_{25}Fe_2Mn$ (747.32): calculated %: C, 19.29; H, 4.856; N, 22.49. Found %: C, 19.74; H, 4.981; N, 22.58. Infrared absorption bands: 3419 cm^{-1}(s) O-H stretch, 2904 (w) C-H stretch, 1597 (vs) C=O stretch, Amide I, 1440 (w), 1313 (w), 1197 (w), 1021 (w), 989 (w), 939 (w), 903 (w), 838 (w), 799 (w), 739 (w), 649 (w).

1.4 X-Ray crystallography

Single-crystal diffraction data of **Fe_2Mn** and **FeMn** were collected at room temperature using a Bruker SMART APEX II CCD diffractometer equipped with a graphite monochromatized MoKα radiation (λ = 0.71073 Å) source. Data reduction was performed using the Bruker SAINT package. Absorption corrections were made with the SADABS program. The structure was solved by direct methods and refined by full-matrix least-squares on F^2 for all non-hydrogen atoms with anisotropic displacement parameters. Hydrogen atoms were introduced in the calculations in accord with the riding model. The CCDC file numbers 2384118 and 2046993 contain the supplementary crystallographic data for these structures. Certain crystallographic data and structural refinement results, and hydrogen bonds are also listed in Tables S1 and S3, respectively.

1.5 Photothermal testing

Aqueous solutions (1.0 mL) of **Fe_2Mn** in a quartz cell at various concentrations (0, 5, 10, 20, 50, 100, 200 $\mu M\cdot L^{-1}$) were irradiated for 10^3 s, with a 730 nm laser at a power density of 1.0 $W\cdot cm^{-2}$. During the irradiation time, the temperature and thermal images of the solutions were recorded with a thermal camera. The photostability of **Fe_2Mn** was also monitored by detecting the changes (if any) in the UV-vis-NIR absorption spectrum in solution before and after irradiation. Subsequently, an aqueous solution of **Fe_2Mn** (200 μM) was irradiated for 10^3 s using a 730 nm laser at a power density of 1.0 $W\cdot cm^{-2}$ with eight heating/cooling cycles under the same conditions.

The photothermal conversion efficiency (η) of the **Fe_2Mn** was calculated according to a standard method.[2] Briefly, under continuous laser irradiation, the temperature of the **Fe_2Mn** aqueous solution was recorded in a time dependent manner, until the solution had reached a steady-state temperature after about 10^3 s. The photothermal conversion efficiency (η) was then calculated according to Equation (1):

$$\eta = \frac{hA(T_{Max} - T_{Surr}) - Q_{Dis}}{I(1 - 10^{-A730})} \quad (1)$$

Where, h represents the heat transfer coefficient. A is the surface area of the quartz sample cell. T_{Max} and T_{Surr} are the maximum steady-state temperature (63.5 °C) and the surrounding temperature of the environment (24.5 °C), respectively. Q_{Dis} is the heat dissipation from the light absorbed by the solvent and the quartz sample cell. I is the incident laser power (1.0 W·cm^{-2}), and $A730$ is the absorbance intensity of the sample at 730 nm (1.394). The value of hA was derived from Equation (2):

$$\tau_s = \frac{m_D * c_D}{h * A} \quad (2)$$

Where τ is the time constant for heat transfer of the system, which was determined to be τ = 234.6 from Figure 4d. The terms m_D and c_D correspond to the mass (1.0 g) and heat capacity (4.2 J/g) of the deionized water used to dissolve the **Fe_2Mn** sample, respectively. So the value hA could be determined as 0.01790 W. Q_{Dis} represents the heat dissipation from the light absorbed by the water and the quartz sample cell; Q_{Dis} was calculated according to Equation (3):

$$Q_{Dis} = \frac{m_D * c_D(T_{Max(water)} - T_{Surr})}{\tau_{s(water)}} \quad (3)$$

Where $T_{max(water)}$ =25.6 °C, T_{Surr} = 24.5 °C, $\tau_{s(water)}$ =345.3. Thus, Q_{Dis} could be calculated as 0.0133 W. According to the data and Equation (1), the photothermal conversion efficiency of the **Fe_2Mn** was determined to be 71.3%.

1.6 Cytotoxicity tests

Mouse breast cancer cells (4T1) and human umbilical vein endothelial cells (HUVEC) were gifts from the Shanghai Proton and Heavy Ion Center and the Institute of Diagnostic and Interventional Radiology, Shanghai Jiao Tong University Affiliated Sixth People's Hospital, Shanghai, China, respectively. Cells were cultured in their corresponding media at 37 °C in a humidified atmosphere of 5% CO_2. Cells were fed every 2 days, and sub-cultured at about 80% confluence.

Biocompatibility was evaluated by means of a standard methyl thiazolyl tetrazolium (MTT) assay. Briefly, 4T1 and HUVEC cells in 100 μL of culture medium were seeded into 96-well plates at a

density of 1 × 10^4 cells per well and grown overnight. Then, the medium was removed, and fresh culture medium containing **Fe_2Mn** at different concentrations (0, 5, 10, 20, 50, 100, 200 μM) was added to the cells, which were continually cultured for 12 h and 24 h at 37 °C under 5 % CO_2. After that, the culture medium was replaced by MTT (100 μL, 0.5 mg·ml^{-1}) and the cells incubated for an additional 4 h at 37 °C. Finally, the MTT was removed, and the resulting formazan crystals were dissolved by adding 100 μL dimethyl sulfoxide. Absorbance measurements were carried out on a Varioskan Flash system at a wavelength of 490 nm.

1.7 Photothermal therapy in vitro

4T1 cells were placed into the wells of a 96-well plate at a cell density of 1×10^4 cells per well such that the cells were fully adhered to the wall and plate transferred to an incubator. Two groups of experiment were set up: One group consisted of 0, 5 μM, 10 μM, 20 μM, 50 μM and 100 μM solutions of **Fe_2Mn**, which were incubated for 12 h. For reference, this will be referred to as the control group. Cell activity was measured by MTT; the other group, termed the experimental group, was incubated for 24 h with the same number of inoculated cells and the same concentration of added **Fe_2Mn** as the previous group. After adding the **Fe_2Mn**, the cells were incubated for 4 h. The cells were then irradiated with a 730 nm 1.0 W·cm^{-2} laser for 5 min and the incubation was continued for 8 h. Finally, cell activity was determined using an MTT assay.

1.8 Confocal laser scanning microscopy

The 4T1 cells were inoculated in confocal dishes and incubated in the incubator until the cells were about to grow all over the confocal dishes. There were two groups, one was a so-called **Fe_2Mn** control group with **Fe_2Mn** concentrations of 0, 20 μM and 100 μM, and the other was, the experimental group with the same solutions as the previous group. Both groups were treated under the same conditions as the photothermal treatment experiments outlined above and monitored by MTT. The cells were stained according to the manufacturer's instructions provided with the MTT kit. Finally, the cells were examined using confocal microscopy. The cells were stained with Calcein AM (green, l_{ex}: 495 nm; l_{em}: 515 nm), which showed green fluorescence for the live cells, and with propidium iodide (PI) (red, l_{ex}: 528 nm; l_{em}: 617 nm), which gave rise to a red fluorescence, for the dead cells.

1.9 Photoacoustic imaging

All in vitro/in vivo photoacoustic experiments were performed with a 3D photoacoustic imaging system, model LOIS-3D, manufactured by TomoWave Laboratories, Suzhou, using a pulsed 730 nm laser at 180 mJ/cm^2.

Tumor models were established through the subcutaneous injection of 100 μL of a cell suspension of 4T1 cells (5 × 10^6 cells) into BALB/c mice in the region of the right hind legs. The BALB/c-nude mice bearing 4T1 tumors were used for PA imaging studies once the tumor reached a volume of 200 mm^3. **Fe_2Mn** was dissolved in phosphate buffered solution (PBS) and injected into the subject mouse via the tail vein at a dose of 25 $\mu mol\cdot kg^{-1}$. Images were analyzed in Image-J by drawing region of interest (ROI) and measuring the signal intensity (SI) of the blood vessel, kidney, liver, tumor, and whole body from the axial and coronal PA images. The normalized PA intensity in the ROI was determined according to the formula: SI_{post}/SI_{pre}, where SI_{pre} and SI_{post} are the signal intensities before and at different times after intravenous injection of the **Fe_2Mn** solution (25 $\mu mol\cdot kg^{-1}$).

1.10 Longitudinal relaxation time T_1 and relaxivity r_1 measurements

The longitudinal relaxation time, T_1, and the relaxivity, r_1, of **Fe_2Mn** were measured with a magnetic resonance imaging (MRI) instrument with a 0.5 T magnetic field (NM120-Analyst). 1 mL aqueous solutions of **Fe_2Mn** at different concentrations (0, 0.1, 0.2, 0.4, 0.6, 0.8 and 1.0) were transferred to clean 2 mL test tubes. The test tubes subject to study were then placed in a special trough device for the T_1 measurements. The T_1 values of **Fe_2Mn** were recorded at different concentrations using the following instrumental parameters: Resonance frequency (SF) = 18 MHz, radio frequency delay time = 0.02 ms, offset frequency (O1) = 537929.43, repeated sampling waiting time (TW) = 8000 ms, spectral width (SW) = 100 kHz, analog gain (RG1) = 20 db, and digital gain (DRG1) = 3. Transverse relaxation times (T_1) were obtained by inversion of the T_1 decay curve. The r_1 value for **Fe_2Mn** was obtained from the slope of the longitudinal relaxivity curve ($1/T_1$) versus the **Fe_2Mn** concentration.

1.11 Molecular Dynamics Simulation

The OPLSS-AA force field[3] and Auxiliary Tools of Force Field (AuToFF) were used to parametrize all C, H, O, N atoms in **Fe_2Mn**, such as the bond parameters, angle parameters and the dihedral angles, and so on. The universal force field (UFF) force field[4] was used to parametrize the Fe^{4+} and Mn^{2+} ions.

The interactions of **Fe_2Mn** and water molecule were simulated by molecular dynamics (MD) simulation. One **Fe_2Mn** molecule and 5560 water molecules were randomly inserted into a cube box with a side length of 6.0 nm. TIP3P was used to model the water molecules.[5] MD simulations were performed using the GROMACS 2021 software package.[6–8] The steepest descent method was applied to minimize the initial energy for each system with a force tolerance of 1 kJ/(mol^{-1} nm^{-1}) and a maximum step size of 0.002 ps before the MD calculations. In all the three directions, periodic boundary conditions were imposed. A leapfrog algorithm was used to

integrate the Newtonian equation of motion.[9] The MD simulation was processed in an NPT isothermal-isobaric ensemble with a simulation time is 20 ns.

In the NPT simulations, the pressure was maintained at 1 bar by the Berendsen barostat in an isotropic manner.[10] The temperature was maintained by the V-rescale thermostat at 298.15 K. The LINCS algorithm was performed for the constrained bond lengths of the hydrogen atoms.[11] The Particle-Mesh-Ewald (PME) with a fourth-order interpolation was used to evaluate the electrostatic interactions, whereas a cutoff of 1.0 nm was employed to calculate the short-range van der Waals interactions.[12]

1.12 MR imaging in vivo

Tumor models were established through the subcutaneous injection of 100 μL of a cell suspension of 4T1 cells (5 × 10^6 cells) into BALB/c mice in the region of the right hind legs. Tumor-bearing mice were used for MR imaging studies once the tumor size reached about 5 mm. **Fe_2Mn** was dissolved in phosphate buffered solution (PBS) and injected into the subject mouse via the tail vein at a dose of 25 μmol·kg^{-1}. Coronal and axial T_1-weighted MR images were recorded on a 3.0 T Siemens MAGNETOM Verio setup. The specific parameters for the T_1-weighted MR imaging in vivo were as follows: slice thickness = 1 mm; repetition time (TR) = 500 ms; echo time (TE) = 15 ms; field of view (FOV) = 36 mm × 52 mm or 31 mm × 51 mm; point resolution = 224 mm × 320 mm or 192 mm × 320 mm. Images were analyzed in Image-J by drawing region of interest (ROI) and measuring the signal intensity (SI) of the kidney, liver, tumor and adjacent muscle of these tissue. We selected adjacent muscle as a reference region for signal-to-background ratios. The relative signal enhancement (RSE%) in kidney, liver and tumor regions was determined according to the formula: $(SI_{post}/SI_{muscle'} - SI_{pre}/SI_{muscle})/SI_{pre}/SI_{muscle}$ × 100%, where SI_{pre} and SI_{post}, SI_{muscle} and $SI_{muscle'}$ are the signal intensities before and at different times after intravenous injection, respectively.

1.13 Photothermal therapy in vivo

Four groups were randomly established from twenty BALB/c mice bearing 4T1 tumors with a volume of 100 mm^3 as follows: "PBS", "Fe_2Mn", "PBS + Laser" and "Fe_2Mn + Laser". Photothermal therapy was initiated when the tumor volumes had reached 100 mm^3. PBS (100 μL) and **Fe_2Mn** (25 μmol/kg) were then injected intravenously into each of ten mice. The mice in the laser groups (five mice per group) were irradiated with a 730 nm laser at a power density of 0.75 W/cm^2 for 5 min at 10 min post-injection. Thermal images of the mice and the temperature increase of the tumor were monitored using a thermal logger. Tumor sizes were determined using a caliper every two days, and the mouse weights were recorded every two days for 14 days.

2. Supplementary Data

2.1 Crystallographic data

Table S1 Crystallographic data for $\mathbf{Fe_2Mn}$ and **FeMn**.

Compound	$\mathbf{Fe_2Mn}$	**FeMn**
Empirical formula	$C_{24}H_{58}Fe_2MnN_{26}O_{25}$	$C_{12}H_{36}FeMnN_{12}O_{18}$
Crystal system	Triclinic	Triclinic
space group	$P\overline{1}$	$P\overline{1}$
Crystal size	0.44 x 0.40x 0.38 mm	0.30 x 0.22x 0.19 mm
a (Å)	9.4538(19)	10.610(7)
b (Å)	12.011(2)	12.022(8)
c (Å)	12.534(3)	12.153(8)
α (deg)	102.214(2)	83.511(7)
β (deg)	111.470(2)	72.918(8)
γ (deg)	103.919(2)	82.244(8)
V, $Å^3$	1212.8(4)	1463.8(17)
Z	1	2
Calculated density, $g·cm^3$	1.749	1.696
F(000)	661	774
R_1 [I>2σ(I)]	0.0424	0.1181
wR_2 [I>2σ(I)]	0.1052	0.2892
R_1(all data)	0.0545	0.1650
wR_2(all data)	0.1125	0.3263
GOF on F^2	1.074	1.065
CCDC number	2384118	2046993

$R1 = \Sigma||F_o| – |F_c||/\Sigma|F_o|$

$wR_2 = [\Sigma w(|F_o^2| – |F_c^2|)^2/\Sigma w(|F_o^2|)^2]^{1/2}$

2.2 Bond valence sums

Table S2 Bond valence sums calculated for $\mathbf{Fe_2Mn}$.

	**Fe1**	**Fe2**	**Mn1**
II	3.66541	3.58654	2.014067
III	4.15065	4.06134	1.842212
IV			1,934048

2.3 Hydrogen bonding data

Table S3. Hydrogen bond distances (Å) and angles (°) for **Fe_2Mn**.

**D – H ··· A**	**D – H / Å**	**H ··· A / Å**	**D ··· A / Å**	**D – H ··· A / °**
O(13)-H(13F)...O(10)#2	0.85	2.62	3.459(15)	167.5
O(13)-H(13F)...O(1)#3	0.85	2.26	2.714(13)	113.7
O(13)-H(13E)...O(4)#4	0.85	2.24	2.822(12)	126.1
O(12)-H(12D)...O(13)	0.85	1.32	1.81(3)	111.5
O(12)-H(12D)...O(1)#3	0.85	2.66	3.135(18)	117.1
O(12)-H(12C)...O(8)#3	0.85	2.28	2.92(3)	133.1
O(11)-H(11D)...O(4)#4	0.85	2.08	2.855(4)	151.1
O(11)-H(11D)...N(8)#4	0.85	2.7	3.284(4)	127.4
O(10)-H(10D)...O(1)#5	0.85	2.17	2.939(5)	150.1
O(10)-H(10D)...N(1)#5	0.85	2.44	3.081(4)	132.3
O(9)-H(9D)...O(10)	0.85	1.87	2.714(5)	170.2
O(9)-H(9C)...O(6)	0.85	2.12	2.939(3)	162.8
O(9)-H(9C)...N(12)	0.85	2.63	3.209(4)	126.4
O(7)-H(7D)...O(2)#5	0.85	1.91	2.751(4)	168.2
N(13)-H(13D)...O(9)	0.85	2.03	2.856(4)	162.4
N(13)-H(13C)...O(5)#1	0.85	1.96	2.798(4)	171.3
N(13)-H(13C)...N(9)#1	0.85	2.65	3.135(4)	118
N(13)-H(13B)...O(3)#6	0.9	1.97	2.846(4)	164.7
N(13)-H(13A)...O(3)#7	0.9	1.98	2.846(4)	160.2
C(11)-H(11A)...O(10)#8	0.97	2.53	3.393(5)	147.9
C(8)-H(8A)...O(2)#3	0.97	2.37	3.343(4)	179.1
C(8)-H(8A)...O(2)#3	0.97	2.37	3.343(4)	179.1
C(11)-H(11A)...O(10)#8	0.97	2.53	3.393(5)	147.9
N(13)-H(13A)...O(3)#7	0.9	1.98	2.846(4)	160.2
N(13)-H(13B)...O(3)#6	0.9	1.97	2.846(4)	164.7
N(13)-H(13C)...N(9)#1	0.85	2.65	3.135(4)	118
N(13)-H(13C)...O(5)#1	0.85	1.96	2.798(4)	171.3
N(13)-H(13D)...O(9)	0.85	2.03	2.856(4)	162.4
O(7)-H(7D)...O(2)#5	0.85	1.91	2.751(4)	168.2
O(9)-H(9C)...N(12)	0.85	2.63	3.209(4)	126.4
O(9)-H(9C)...O(6)	0.85	2.12	2.939(3)	162.8
O(9)-H(9D)...O(10)	0.85	1.87	2.714(5)	170.2
O(10)-H(10D)...N(1)#5	0.85	2.44	3.081(4)	132.3
O(10)-H(10D)...O(1)#5	0.85	2.17	2.939(5)	150.1

O(11)-H(11D)...N(8)#4	0.85	2.7	3.284(4)	127.4
O(11)-H(11D)...O(4)#4	0.85	2.08	2.855(4)	151.1
O(12)-H(12C)...O(8)#3	0.85	2.28	2.92(3)	133.1
O(12)-H(12D)...O(1)#3	0.85	2.66	3.135(18)	117.1
O(12)-H(12D)...O(13)	0.85	1.32	1.81(3)	111.5
O(13)-H(13E)...O(4)#4	0.85	2.24	2.822(12)	126.1
O(13)-H(13F)...O(1)#3	0.85	2.26	2.714(13)	113.7
O(13)-H(13F)...O(10)#2	0.85	2.62	3.459(15)	167.5
C(8)-H(8A)...O(2)#3	0.97	2.37	3.343(4)	179.1
C(11)-H(11A)...O(10)#8	0.97	2.53	3.393(5)	147.9
N(13)-H(13A)...O(3)#7	0.9	1.98	2.846(4)	160.2
N(13)-H(13B)...O(3)#6	0.9	1.97	2.846(4)	164.7
N(13)-H(13C)...N(9)#1	0.85	2.65	3.135(4)	118
N(13)-H(13C)...O(5)#1	0.85	1.96	2.798(4)	171.3
N(13)-H(13D)...O(9)	0.85	2.03	2.856(4)	162.4
O(7)-H(7D)...O(2)#5	0.85	1.91	2.751(4)	168.2
O(9)-H(9C)...N(12)	0.85	2.63	3.209(4)	126.4
O(9)-H(9C)...O(6)	0.85	2.12	2.939(3)	162.8
O(9)-H(9D)...O(10)	0.85	1.87	2.714(5)	170.2
O(10)-H(10D)...N(1)#5	0.85	2.44	3.081(4)	132.3
O(10)-H(10D)...O(1)#5	0.85	2.17	2.939(5)	150.1
O(11)-H(11D)...N(8)#4	0.85	2.7	3.284(4)	127.4
O(11)-H(11D)...O(4)#4	0.85	2.08	2.855(4)	151.1
O(12)-H(12C)...O(8)#3	0.85	2.28	2.92(3)	133.1
O(12)-H(12D)...O(1)#3	0.85	2.66	3.135(18)	117.1
O(12)-H(12D)...O(13)	0.85	1.32	1.81(3)	111.5
O(13)-H(13E)...O(4)#4	0.85	2.24	2.822(12)	126.1
O(13)-H(13F)...O(1)#3	0.85	2.26	2.714(13)	113.7
O(13)-H(13F)...O(10)#2	0.85	2.62	3.459(15)	167.5

Symmetry codes: #1: -x,-y+1,-z+1; #2: x+1,y,z; #3: -x+1,-y,-z+1; #4: x,y,z-1; #5: -x,-y,-z+1; #6: x-1,y,z-1; #7: -x+1,-y+1,-z+1; #8: x,y,z+1.

2.4 Hematological analysis

Table S4. Hematological analysis of blood samples from healthy mice after intravenous injection of **Fe_2Mn** (25 μmol/kg).

	Baseline	24h	48h	*p* value	Reference range	Statistical test
**White blood cell (×10^9/L)**	1.0(±0.6)	1.4(±1.4)	1.8(±0.9)	0.65	0.8-10.6	ANOVA
**Lymphocytes (×10^9/L)**	0.8(±0.7)	0.7(±0.9)	0.9(±0.8)	0.90	0.6-8.9	ANOVA
**Monocytes (×10^9/L)**	0.0(±0.0)	0.1(±0.1)	0.1(±0.1)	0.11	0.04-1.4	ANOVA
**Neutrophils (×10^9/L)**	0.1(±0.1)	0.5(±0.6)	0.7(±0.3)	0.25	0.23-3.6	ANOVA
**Red blood cells (×10^{12}/L)**	5.8(±1.7)	6.5(±0.5)	6.6(±0.7)	0.70	6.5-11.5	ANOVA
**Haemoglobin (g/L)**	106.7(±31.2)	132.0(±15.7)	128.7(±18.8)	0.46	110-165	ANOVA
**Hematocrit (%)**	24.9(±7.0)	31.1(±1.9)	31.4(±2.6)	0.25	35-55	ANOVA
**Mean cell volume (fL)**	43.0(±1.9)	48.3(±1.0)	47.9(±1.8)	0.01	41-55	ANOVA
**Mean corpuscular haemoglobin (pg)**	18.2(±1.2)	20.5(±2.8)	19.5(±1.9)	0.50	13-18	ANOVA
**Mean corpuscular haemoglobin concentration (g/L)**	427.0(±46.9)	424.3(±50.6)	410.0(±51.2)	0.91	300-360	ANOVA
**Red cell distribution width (%)**	16.0(±0.7)	16.0(±2.3)	16.7(±1.7)	0.75	12-19	ANOVA
**Platelets (×10^9/L)**	447.3(±334.5)	450.3(±315.5)	456.0(±174.7)	0.65	400-1600	ANOVA
**Mean platelet volume (fL)**	5.2(±0.3)	5.9(±0.6)	5.5(±0.1)	0.36	4.0-6.2	ANOVA
**Platelet distribution width (%)**	16.8(±0.8)	17.7(±0.9)	16.8(±0.6)	0.35	12.0-17.5	ANOVA

Statistical analysis was carried out using SPSS (latest version). Initial testing for normality was carried out, and where data were parametric. A repeated analysis of variance (ANOVA) between the test samples was employed, adopting a 5% level of significance.

Hematological changes following **Fe_2Mn** solution being injected into the tail vein of mice are presented in the table above. The following exhibited statistically significant increase was mean cell volume (p = 0.01). No changes were reported in other blood indicators ($p > 0.05$).

2.5 Photothermal conversion efficiency

Table S5 Photothermal conversion efficiencies of **Fe_2Mn** and different photothermal reagents

Category	Type	Name	Laser	*η*/%	Ref
Inorganic materials	Gold nanoparticle	Anionic-AuNRs	808	32	
	Platinum nanoparticle	mPt@mSiO_2-GdDTPA	808	27	[14]
	Carbon material	Ta_4C_3-SP	808	44.7	[15]
	dichalcogenides	Ni_9S_8 NPs	1064	46	[16]
	Oxide nanoparticle	Bio-MnO_2 NPs	808	44	[17]
Organic materials	Indocyanine green	ICG	808	3.37	[18]
	diimine	P(QDI)	808	64.7	[19]
	amidoamine	DSP	808	44.2	[20]
	Cryptocyanine	Mito-CCy	730	9.5	[21]
Conjugated polymer	Phenyl-benzobisthiazole	PPBBT	808	32.4	[22]
	polypyrrole	PPy@FeOCl	1064	64.6	[23]
Metal complex	Porphyrin-based	Por-DPP NPs	808	62.5	[24]
**This work**	Fe(IV)-based	**Fe_2Mn**	730	71.37	

2.6 Hydrogen bond interactions seen in the solid state

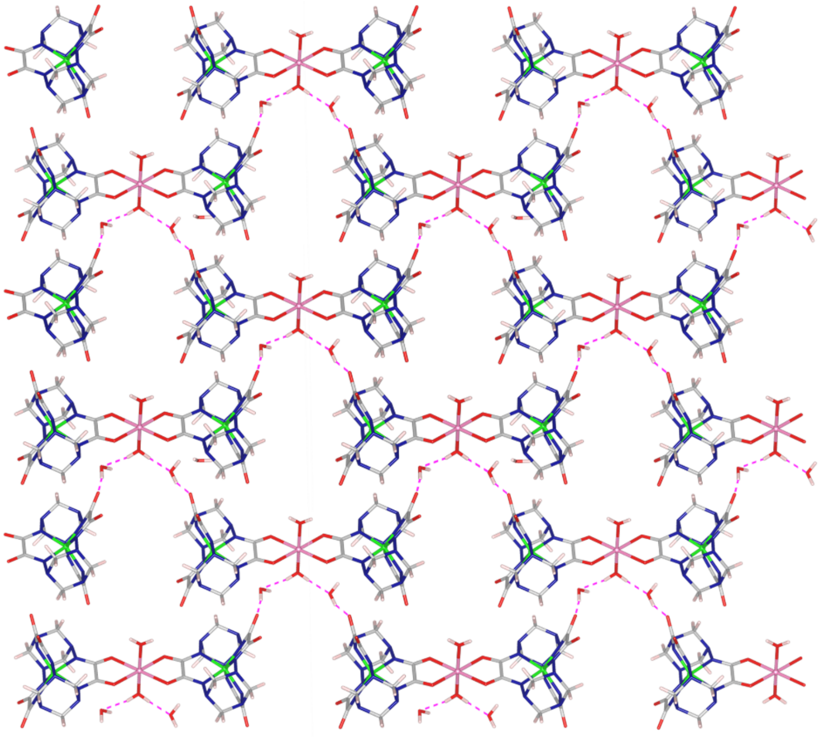

Figure S1. Hydrogen bonds between Fe_2Mn and water molecules seen in the solid state. (Dashed lines represent hydrogen bonds inferred on the basis of the metric parameters.)

2.7 Photographs of samples in aqueous solution

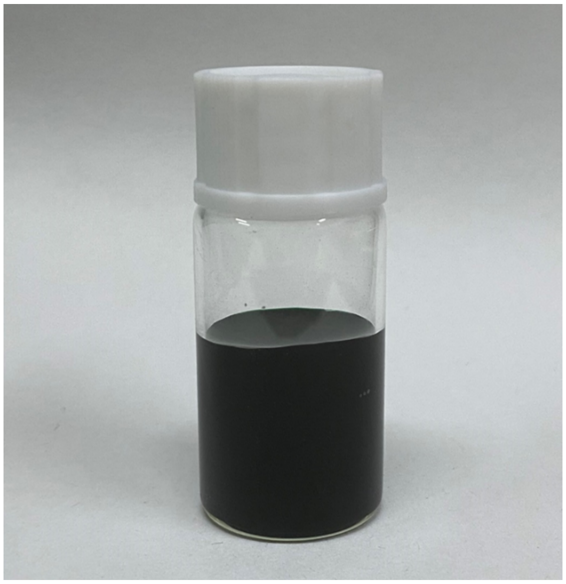

Figure S2. Photograph of Fe_2Mn (5 mM) in aqueous solution.

2.8 Distance parameters and size calculation for Fe_2Mn

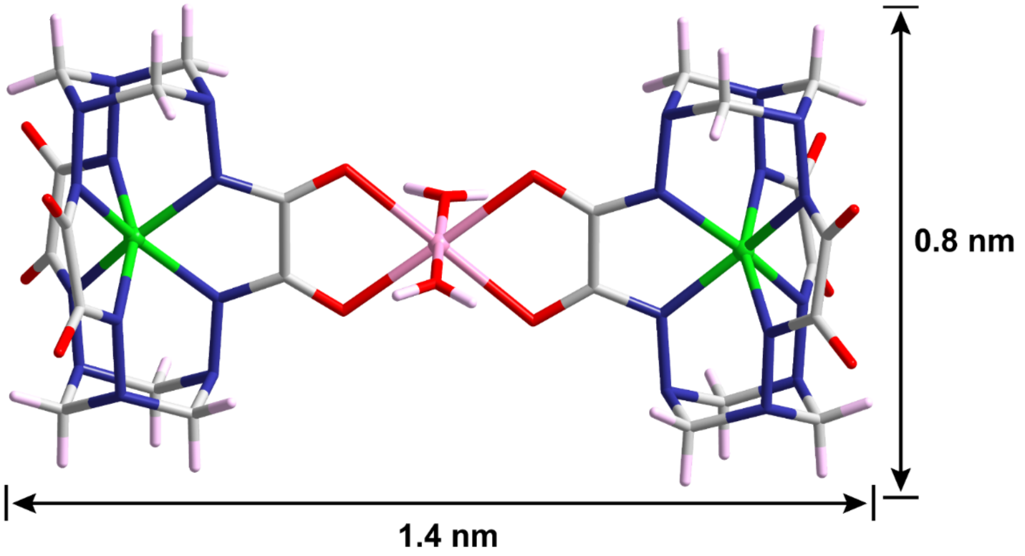

Figure S3. Calculation used to size **Fe_2Mn**.

2.9 MR relaxivity rate measurements for Fe_2Mn

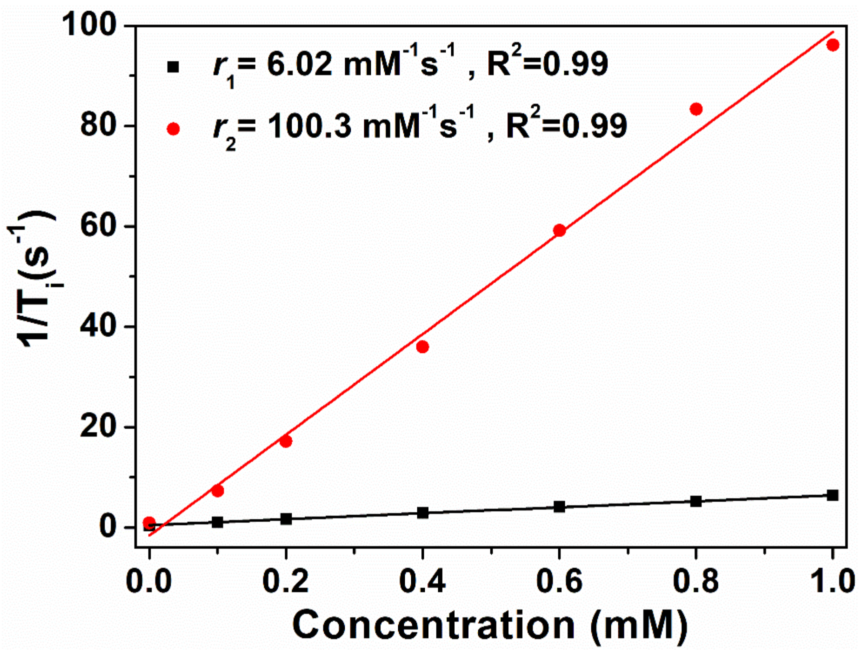

Figure S4. MR relaxation rate versus concentration of **Fe_2Mn** under a 3.0 T applied magnetic field at 25 °C.

2.10 MR relaxivity rate measurements of Fe-HDCL

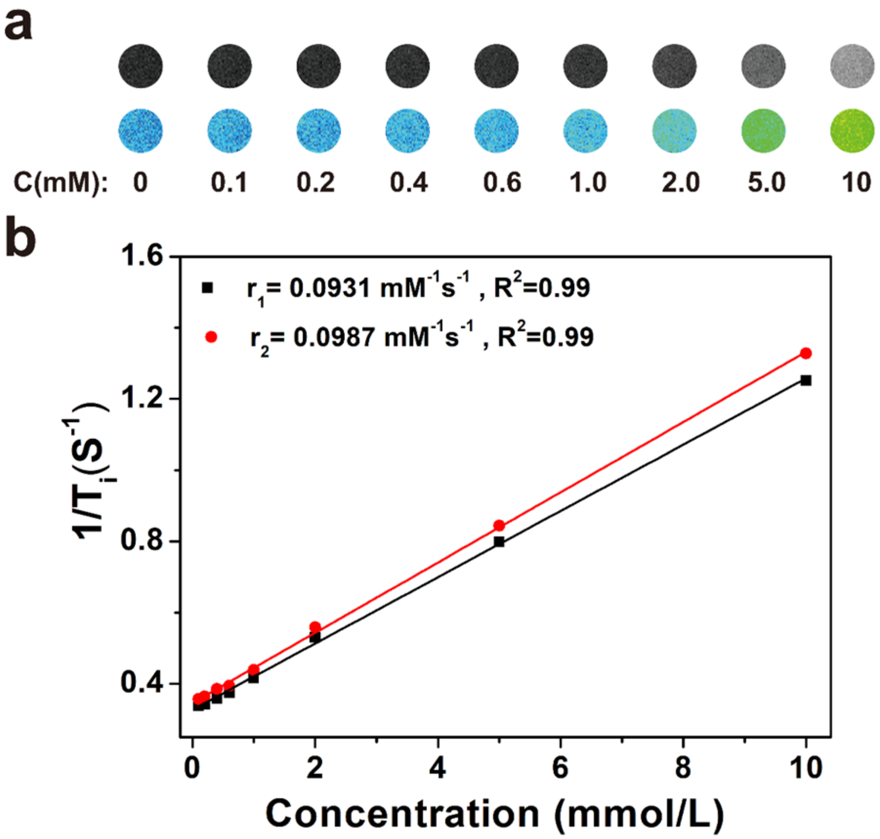

Figure S5. (a) T_1-weighted and color-mapped phantom MR images for aqueous solutions containing various concentrations of Fe-HDCL at 0.5 T, 25 °C. (b) MR relaxation rate versus concentrations of Fe-HDCL under an applied 0.5 T magnetic field at 25 °C.

2.11 MR relaxivity rate measurements for $MnCl_2$

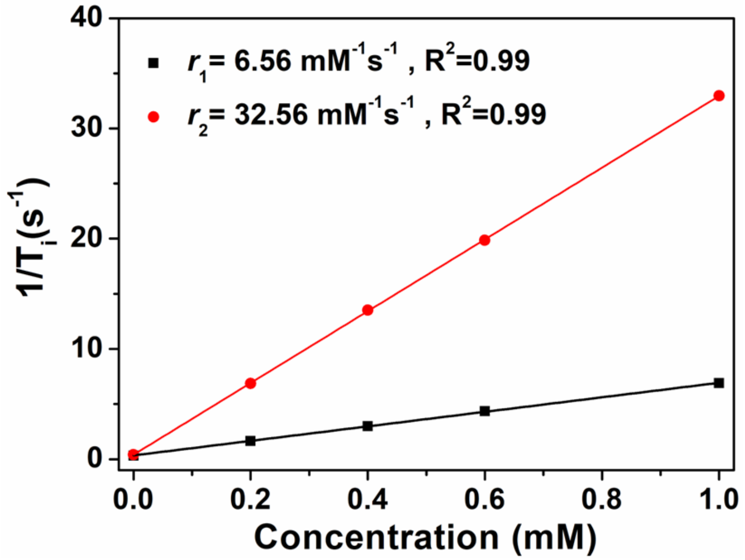

Figure S6. MR relaxation rate versus concentrations of $MnCl_2$ under an applied 0.5 T magnetic field at 25 °C.

2.12 Interactions between Fe_2Mn and water molecules in the solid state

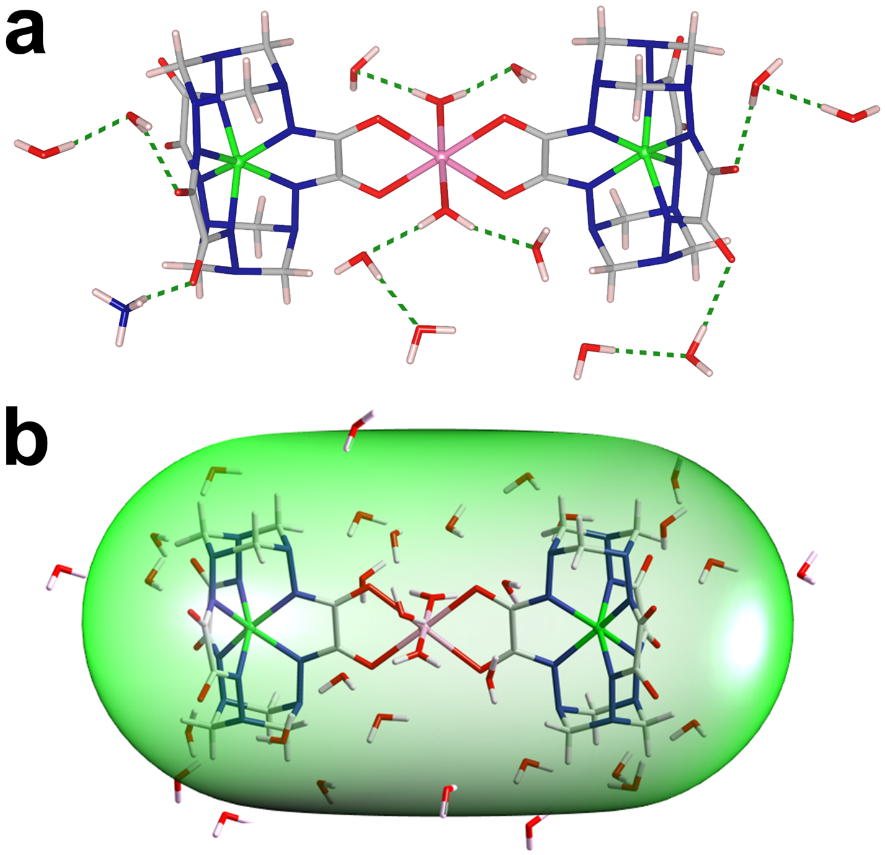

Figure S7. (a) Hydrogen bond interactions between **Fe_2Mn** and adjacent water molecules seen in the solid state. (b) View of the second sphere wrapped around the full **Fe_2Mn** nanocluster highlighting the high concentration of water molecules within the defined volume.

2.13 Solid-state FT-IR spectra of Fe_2Mn.

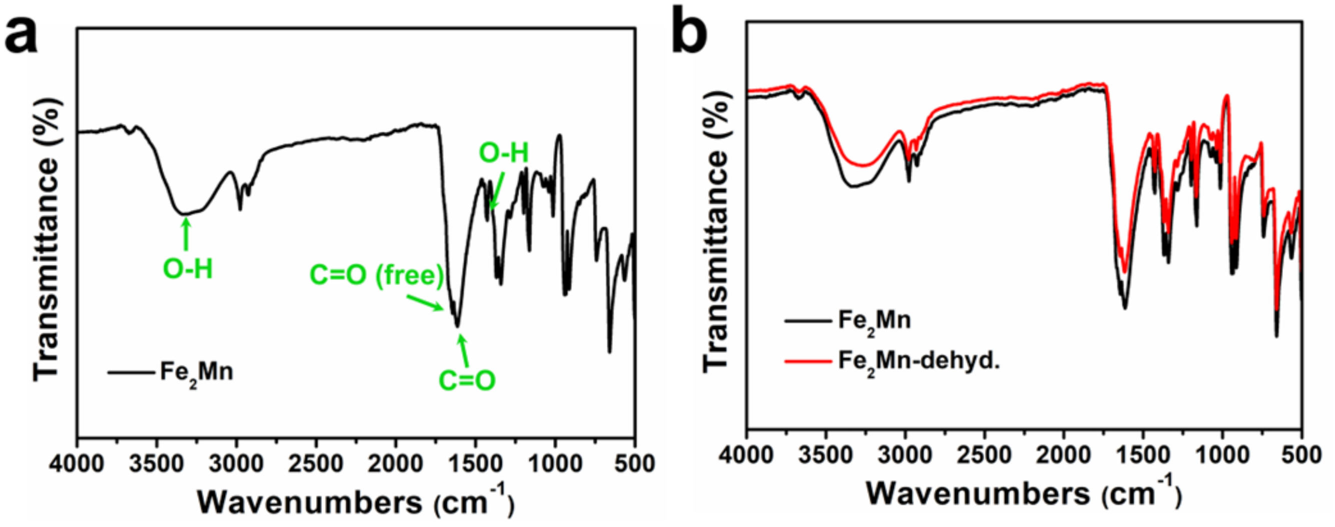

Figure S8. (a) Solid-state FT-IR spectra of crystalline **Fe_2Mn**. (b) Solid-state FT-IR spectra of crystalline **Fe_2Mn** and dehydrated **Fe_2Mn**.

2.14 MR relaxivity rate measurements for FeMn

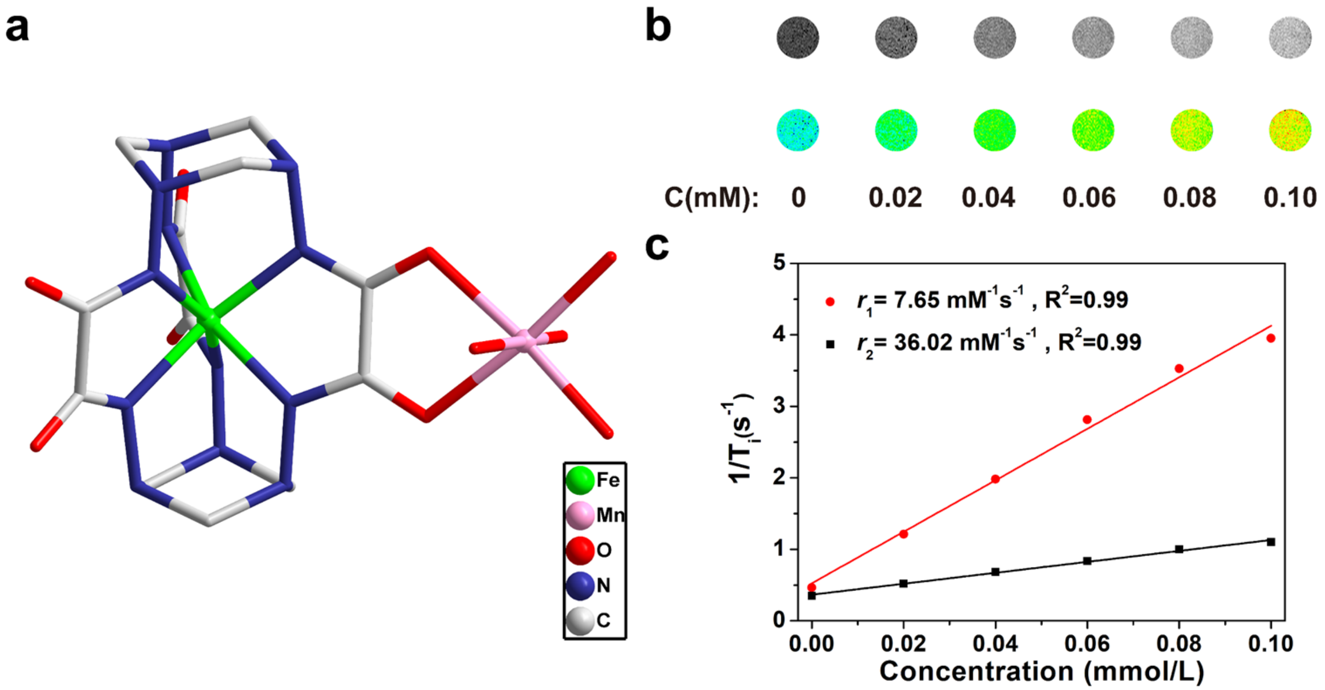

Figure S9. (a) Molecular structure of **FeMn**. Hydrogen atoms and solvent molecules are omitted for clearly. (b) T_1-weighted and color-mapped phantom MR images for aqueous solutions containing various concentrations of **FeMn** at 0.5 T, 25 °C. (c) MR relaxation rate versus concentrations of **FeMn** under an applied 0.5 T magnetic field at 25 °C.

2.15 Stability comparison of FeMn in aqueous solution

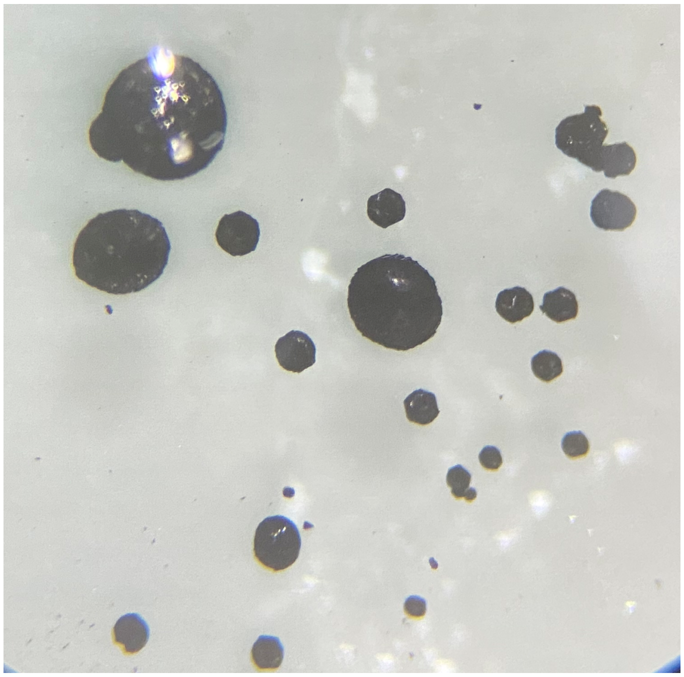

Figure S10. Photograph of precipitates were formed within 24 hours when **FeMn** was dissolved in water, demonstrating poor thermodynamic stability.

2.16 Long-term solubility of Fe_2Mn in aqueous solution.

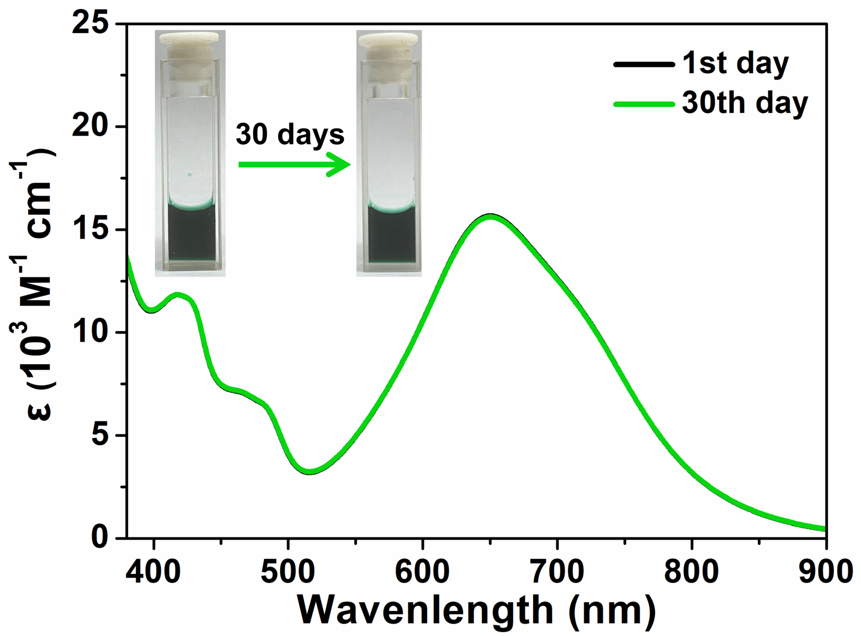

Figure S11. UV-vis-NIR absorption spectra of 500 μM aqueous solution of **Fe_2Mn** after 30 days. **Fe_2Mn** remains fully dissolved and clear in water for >30 days, as confirmed by the absence of precipitates in the photograph of 500 μM **Fe_2Mn** solution (left panel). This stability is attributed to its superhydrophilic architecture, para-positioned water coordination sites, and strong chelating interactions between Mn(II) and Fe-HDCL subunits.

2.17 Relaxivity measurements in various media

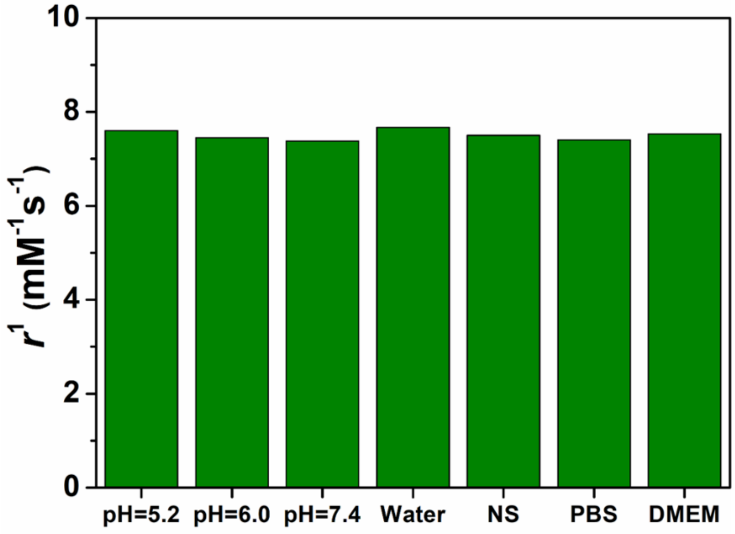

Figure S12. Relaxivity measurement of **Fe_2Mn** incubated in deionized water and other aqueous media after 48 h. Bicarbonate buffer (pH = 5.2, 6.0, and 7.4, respectively); deionized water (water in the inset); normal saline (NS); phosphate buffered saline (PBS); Dulbecco's modified eagle medium (DMEM).

2.18 Free manganese(II) ion measurements with Erichrome Black T.

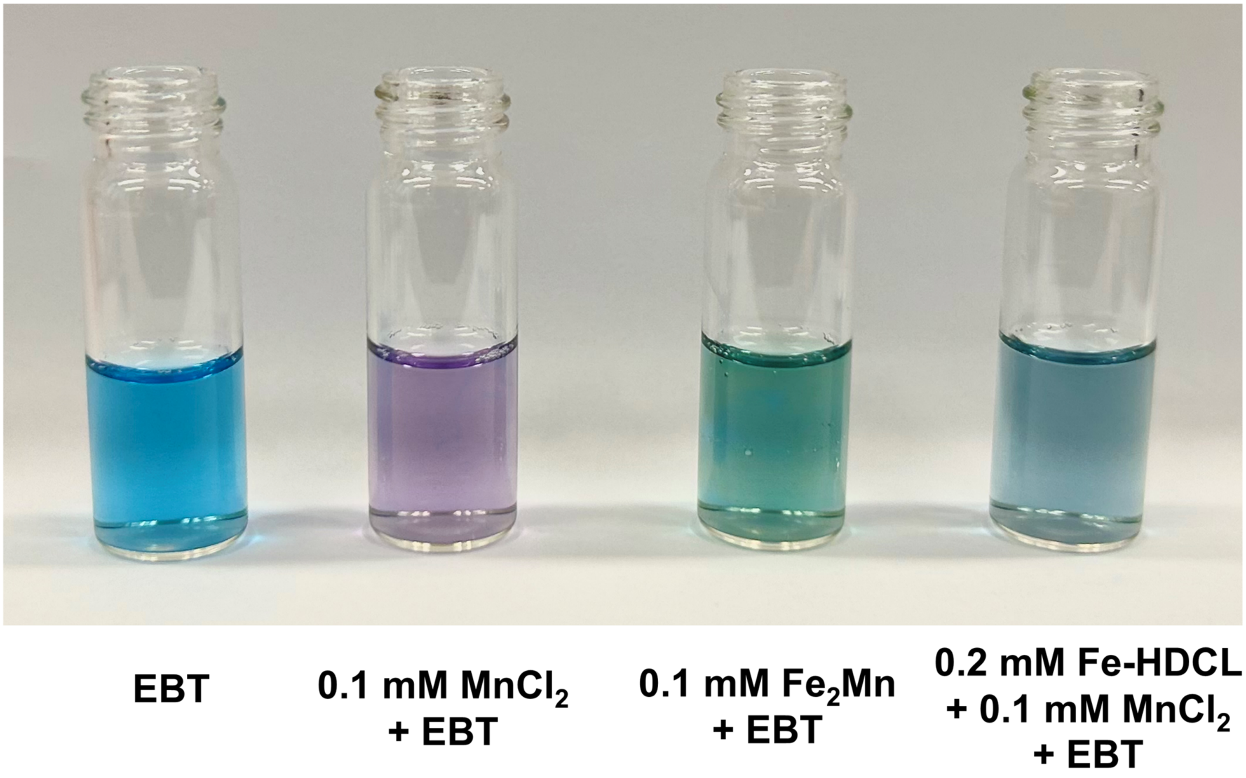

Figure S13. Measurements of free manganese(II) ion in aqueous solutions of **Fe_2Mn** as determined using the Eriochrome Black T (EBT) assay.

2.19 Cell viability studies involving Fe_2Mn

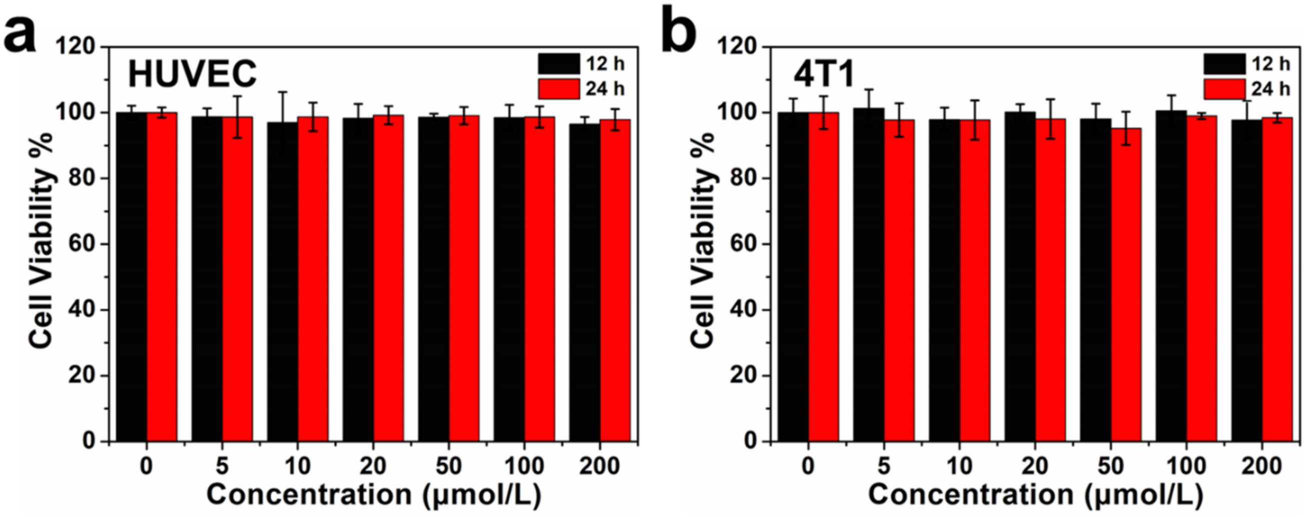

Figure S14. Cell viability of 4T1 cells (a) and HUVEC cells (b) 12 and 24 h after incubation with various concentrations of **Fe_2Mn**.

2.20 Cell viability studies for $MnCl_2$

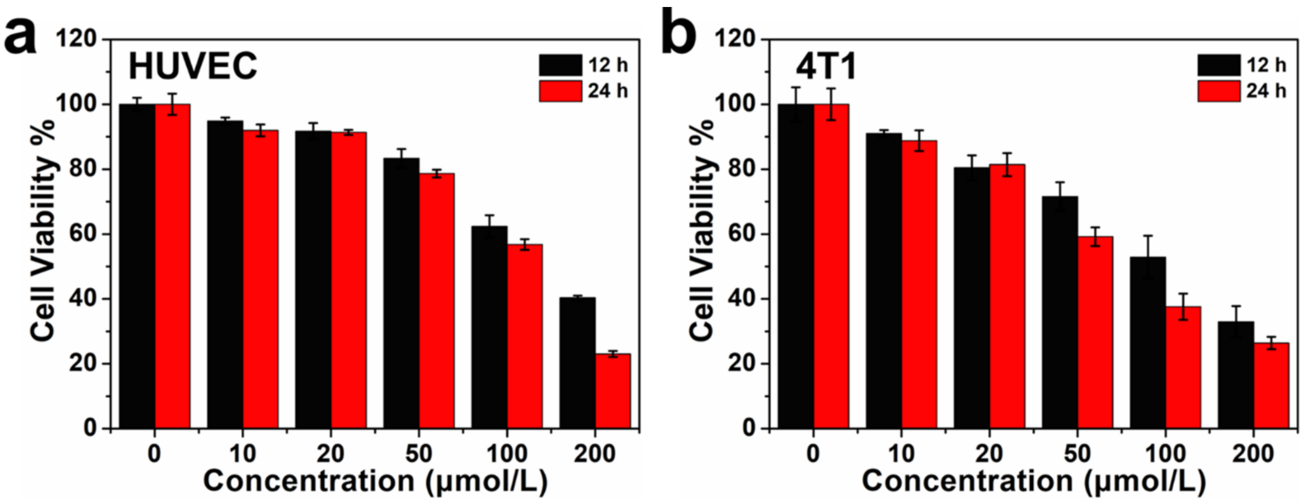

Figure S15. Cell viability of 4T1 cells (a) and HUVEC cells (b) 12 and 24 h after incubation with various concentrations of **$MnCl_2$**.

2.21 Biological clearance studies

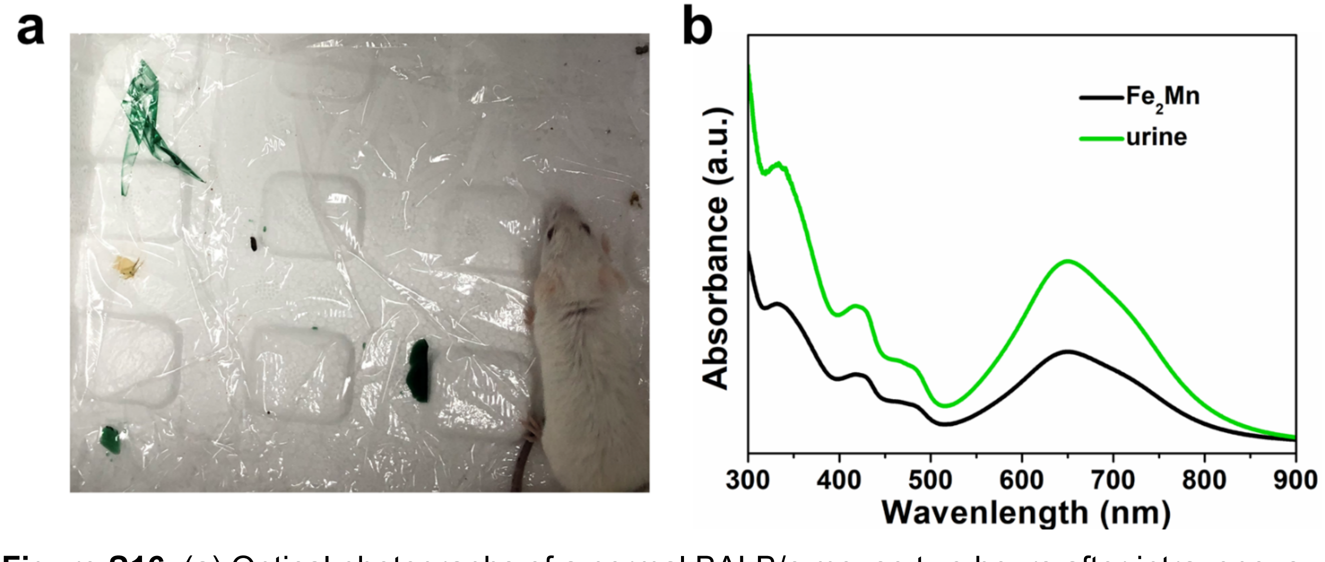

Figure S16. (a) Optical photographs of a normal BALB/c mouse two hours after intravenous injection of **Fe_2Mn** (25 μmol/kg). (b) UV-vis-NIR absorption spectra of the mouse urine taken from the animals in (a) and an aqueous solution of **Fe_2Mn**.

2.22 Biodistribution studies

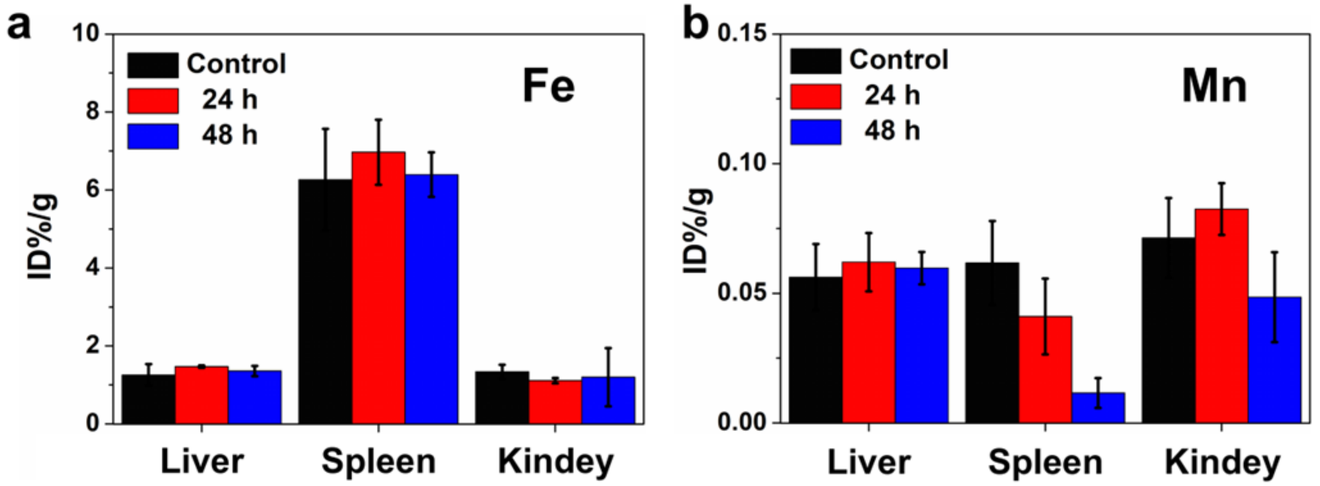

Figure S17. Biodistribution of **Fe_2Mn** in 4T1 tumor-bearing mice at 24 h and 48 h post injection. The concentrations of Fe and Mn in various tissues were determined by ICP-AES. Different organs including liver, spleen, kidney, and heart, were examined.

2.23 Pathological examination

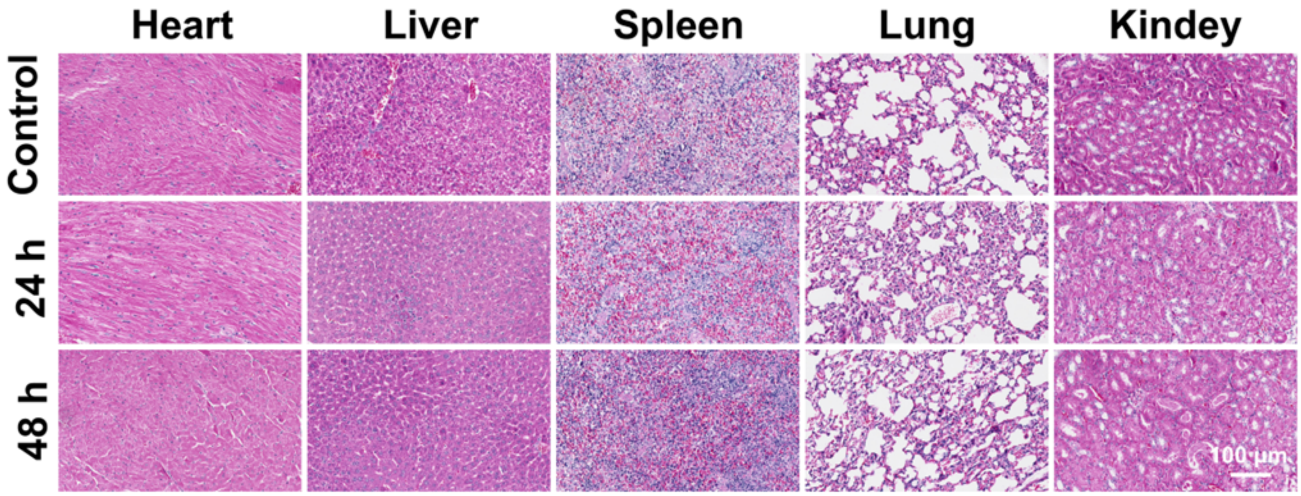

Figure S18. Pathological examination by H&E staining showing images of major organs collected from healthy mice.

2.24 MR images of an implanted murine tumor

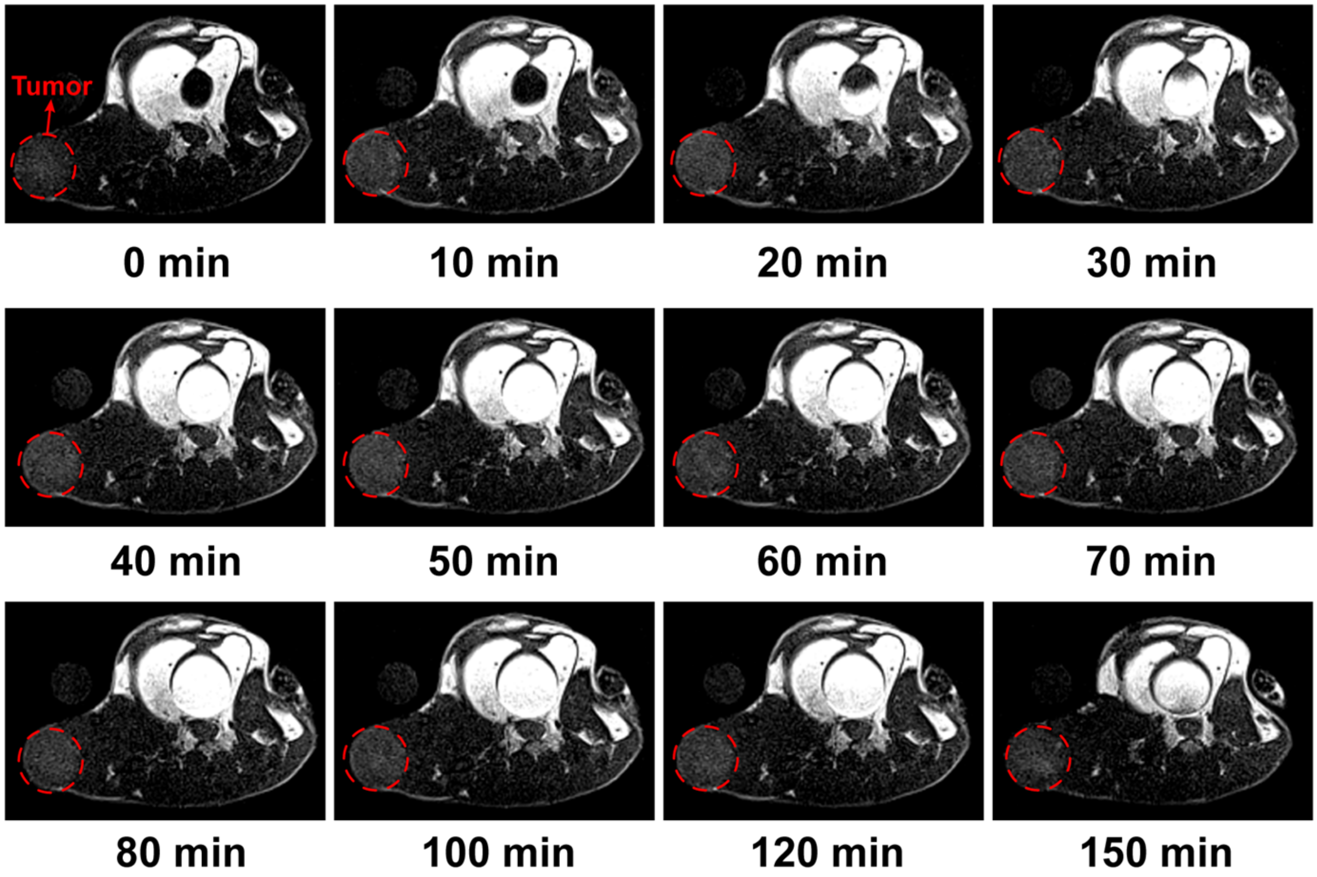

Figure S19. Axial T_1-weighted MR images of a BALB/c mouse bearing 4T1 tumors before and at different times after subjecting to intravenous injection with **Fe_2Mn** (25 μmol/kg).

2.25 Photographs of crystalline samples

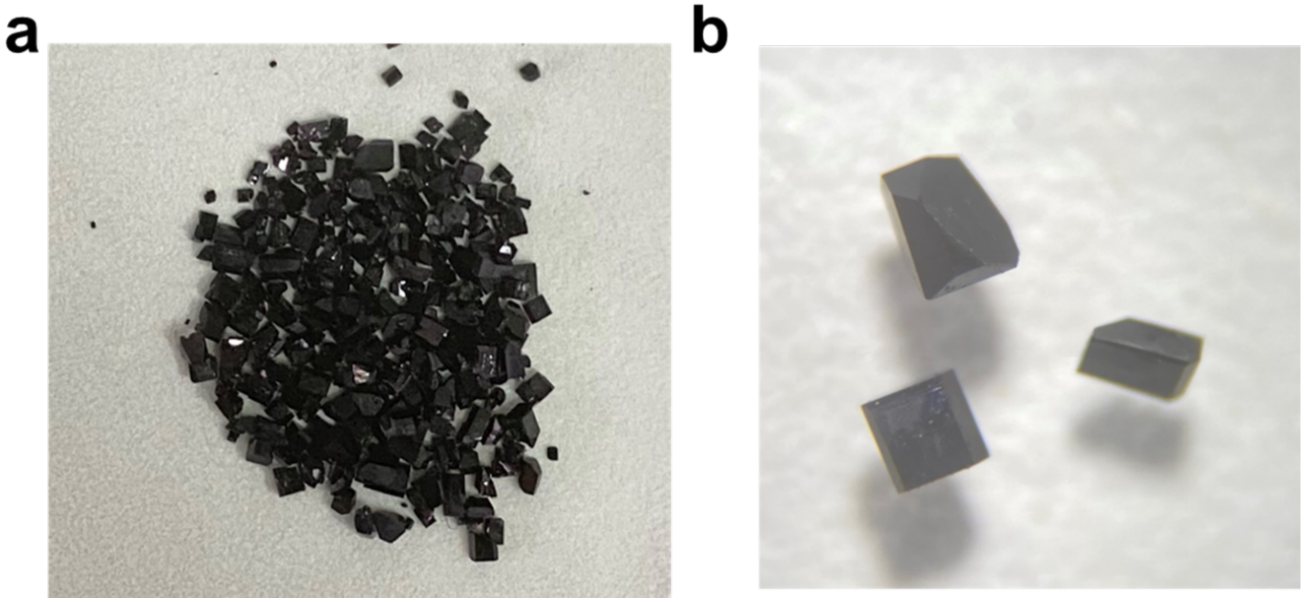

Figure S20. Photographs of crystalline samples of **Fe_2Mn**.

2.26 UV-vis-NIR absorption spectrum of a powder sample of Fe_2Mn

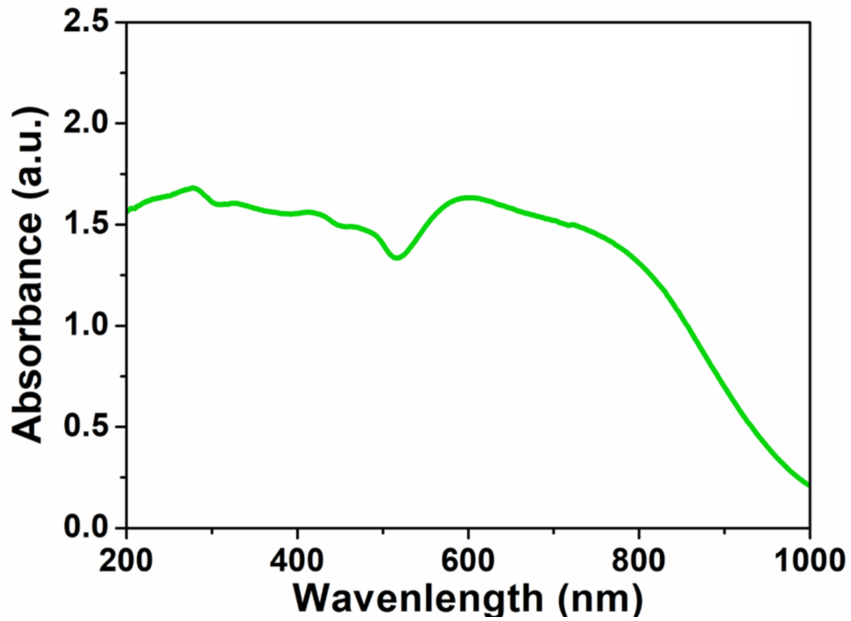

Figure S21. UV-vis-NIR absorption spectrum of $\mathbf{Fe_2Mn}$ in the solid state.

2.27 UV-vis-NIR spectrum of Fe_2Mn vs the solar spectrum

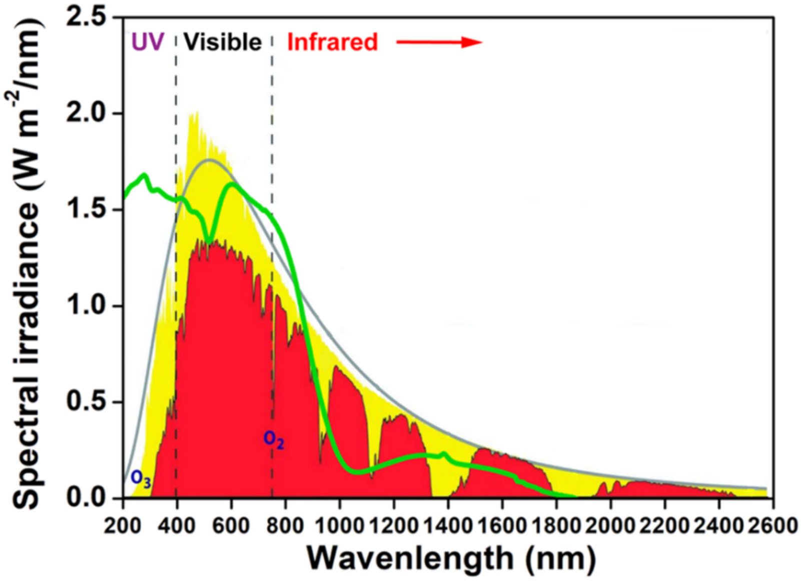

Figure S22. UV-vis-NIR spectrum of $\mathbf{Fe_2Mn}$ overlaid with the irradiation spectrum of sunlight. This figure shows the radiation spectrum for the direct light both at the top of the earth's atmosphere (yellow) and at sea level (red).[25]

2.28 Photothermal studies

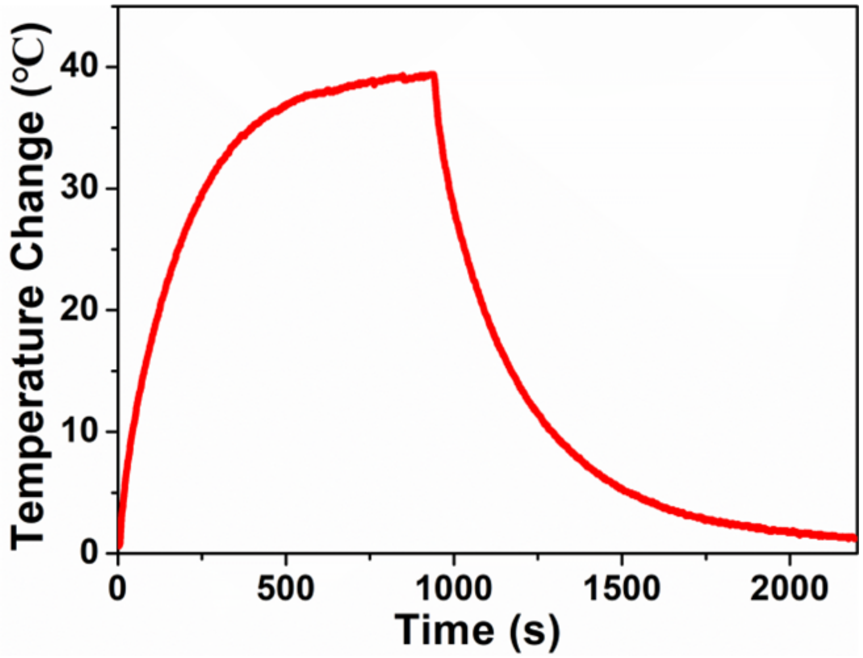

Figure S23. Photothermal effect produced via the irradiation of an aqueous solution (200 μM) with a NIR laser followed by the temperature change observed after the laser was shut off.

2.29 Stability testing

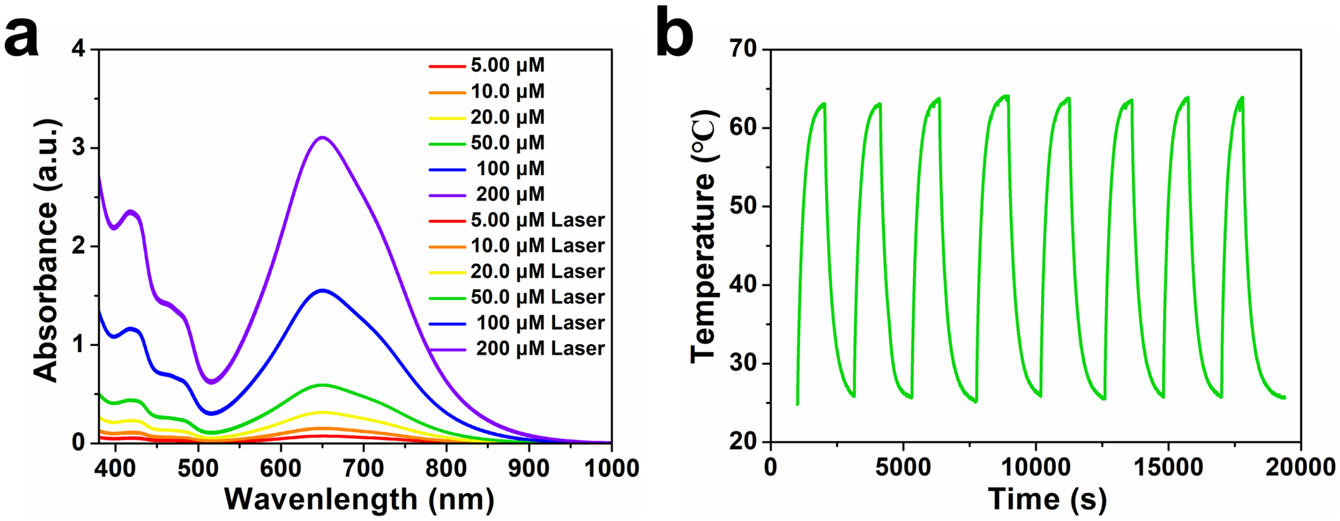

Figure S24. (a) UV-vis-NIR absorption spectra of **Fe_2Mn** recorded at different concentrations before and after 730 nm photoirradiation for 10^3 s at a power density of 1.0 W/cm^2. (b)ON/OFF cycles for aqueous solutions of **Fe_2Mn** (200 μM) subject to photoirradiation (730 nm, 1.0 W/cm^2).

3. Supplementary references

[1] Y. Xu, C. Li, Z.-Y. Yu, J. Wang, Z. Liu, C. Jiang, Z.-F. Chen, X. Wu, M.-X. Li, H. Yang, Z.-X. Wang, J. L. Sessler, *CCS Chem.* **2024**, *6*, 1184–1197.

[2] D. Kieth Roper, W. Ahn, M. Hoepfner, *J. Phys. Chem. C* **2007**, *111*, 3636–3641.

[3] W. L. Jorgensen, D. S. Maxwell, J. Tirado-Rives, *J. Am. Chem. Soc.* **1996**, *118*, 11225–11236.

[4] A. K. Rappe, C. J. Casewit, K. S. Colwell, W. A. Goddard, W. M. Skiff, *J. Am. Chem. Soc.* **1992**, *114*, 10024–10035.

[5] W. L. Jorgensen, J. Chandrasekhar, J. D. Madura, R. W. Impey, M. L. Klein, *J. Chem. Phy.* **1983**, *79*, 926–935.

[6] D. Van Der Spoel, E. Lindahl, B. Hess, G. Groenhof, A. E. Mark, H. J. C. Berendsen, *J. Comput. Chem.* **2005**, *26*, 1701–1718.

[7] M. J. Abraham, T. Murtola, R. Schulz, S. Páll, J. C. Smith, B. Hess, E. Lindahl, *SoftwareX* **2015**, *1–2*, 19–25.

[8] H. J. C. Berendsen, D. Van Der Spoel, R. Van Drunen, *Comput. Phys. Commun.* **1995**, *91*, 43–56.

[9] W. F. Van Gunsteren, H. J. C. Berendsen, *Mol. Simulat.* **1988**, *1*, 173–185.

10] H. J. C. Berendsen, J. P. M. Postma, W. F. Van Gunsteren, A. DiNola, J. R. Haak, *J. Chem. Phy.* **1984**, *81*, 3684–3690.

[11] B. Hess, H. Bekker, H. J. C. Berendsen, J. G. E. M. Fraaije, *J. Comput. Chem.* **1997**, *18*, 1463–1472.

[12] T. Darden, D. York, L. Pedersen, *J. Comput. Chem.***1993**, *98*, 10089–10092.

[13] L. An, Y. Wang, J. Lin, Q. Tian, Y. Xie, J. Hu, S. Yang, *ACS Appl. Mater. Interfaces* **2019**, *11*, 15251–15261.

[14] L. Zhao, X. Ge, G. Yan, X. Wang, P. Hu, L. Shi, O. S. Wolfbeis, H. Zhang, L. Sun, *Nanoscale* **2017**, *9*, 16012–16023.

[15] H. Lin, Y. Wang, S. Gao, Y. Chen, J. Shi, *Adv. Mater.* **2018**, *30*, 1703284.

[16] Z. Lei, W. Zhang, B. Li, G. Guan, X. Huang, X. Peng, R. Zou, J. Hu, *Nanoscale* **2019**, *11*, 20161–20170.

[17] J. Liu, L. Feng, Y. Wu, *Nanoscale* **2021**, *13*, 11093–11103.

[18] H.-J. Yoon, H.-S. Lee, J.-Y. Lim, J.-H. Park, *ACS Appl. Mater. Interfaces* **2017**, *9*, 5683–5691.

[19] C. Liu, S. Zhang, J. Li, J. Wei, K. Müllen, M. Yin, *Angew. Chem. Int. Ed.* **2019**, *58*, 1638–1642.

[20] Y. Lyu, D. Cui, H. Sun, Y. Miao, H. Duan, K. Pu, *Angew. Chem. Int. Ed.* **2017**, *56*, 9155–9159.

[21] H. S. Jung, J.-H. Lee, K. Kim, S. Koo, P. Verwilst, J. L. Sessler, C. Kang, J. S. Kim, *J. Am. Chem. Soc.* **2017**, *139*, 9972–9978.

[22] P. Chen, Y. Ma, Z. Zheng, C. Wu, Y. Wang, G. Liang, *Nat. Commun.* **2019**, *10*, 1192.

[23] X. Wang, Y. Ma, X. Sheng, Y. Wang, H. Xu, *Nano Lett.* **2018**, *18*, 2217–2225.

[24] F. Wu, L. Chen, L. Yue, K. Wang, K. Cheng, J. Chen, X. Luo, T. Zhang, *ACS Appl. Mater. Interfaces* **2019**, *11*, 21408–21416.

[25] Y. Tanaka, K. Matsuo, S. Yuzuriha, *Eplasty* **2010**, *10*, 327-335.
